# The influence of car traffic on airborne fungal diversity in Tianjin, China

**DOI:** 10.1080/21501203.2023.2300343

**Published:** 2024-01-18

**Authors:** Mohammed H.M. Muafa, Ziwei M. Quach, Amran A.Q.A. Al-Shaarani, Md M.H. Nafis, Lorenzo Pecoraro

**Affiliations:** School of Pharmaceutical Science and Technology, Tianjin University, Tianjin, China

**Keywords:** Airborne fungal communities, concentration, diversity, environmental factors, intensity of car traffic, urban environments

## Abstract

Little is known about the effect of car traffic on airborne fungal communities. We investigated the environmental factors affecting the diversity and concentration of airborne fungi at high-traffic density junctions, in Tianjin, China. A total of 244 fungal strains belonging to 78 species and 45 genera of Ascomycota (78.69%) and Basidiomycota (21.31%) were isolated and identified using morphological and molecular analysis. *Aspergillus* was the species-richest genus, with 9 recorded species, followed by *Alternaria* and *Cladosporium*, both with 8 species. *Coprinellus radians* was the most abundant fungal species, with 31 isolated strains, followed by *Alternaria alternata* (26 strains), *Cladosporium cladosporioides* (21), *Alternaria compacta* (13), and *Cladosporium tenuissimum* (11). We found a higher diversity and concentration of airborne fungi in the analysed urban air environments when the road traffic was at its highest intensity. Higher level of car traffic resulted in higher concentrations of fungal particles in the air for various taxa, including *Alternaria*, *Aspergillus*, and *Cladosporium*, which are known to cause respiratory allergies and infections. This result suggests that reducing vehicular traffic could be an effective measure to control airborne fungal exposure and microbial pollution.

## 1. Introduction

Microbial particles, such as fungi, bacteria, and allergens, are common natural components of the air (Kim et al. 2018; Lu et al. 2022; Yuan et al. 2022; Nageen et al. 2023). In particular, fungi represent a large portion of the airborne microbes, since they are among the most abundant, widely dispersed, and pervasive organisms on Earth (Wu et al. 2000; Nageen et al. 2021). Indeed, fungi are ubiquitous in nature, playing different roles in the environment as symbionts, saprotrophs, or parasites, which enables them to colonise diverse habitats (Cvetnić and Pepeljnjak 1997; Anees-Hill et al. 2022; Martins 2022). Fungal organisms are abundant in the environment, accounting for roughly 25% of global biomass (Miller 1992; Sorenson 1999; Nageen et al. 2023). Spores of different fungal species are dispersed in the atmosphere, and they are considered to be linked to air pollution (Shelton et al. 2002), thus affecting human health (Sivagnanasundaram et al. 2019; Nafis et al. 2023). Bioaerosols containing airborne microorganisms and their waste products can cause respiratory disorders and other adverse health effects, such as hypersensitivity pneumonitis and toxic responses (Harrison et al. 1992; Hargreaves et al. 2003; Fracchia et al. 2006; Cai et al. 2019). Exposure to high concentrations of airborne microbes has been linked to asthma and rhinitis (Beaumont 1988; Baxi et al. 2016), hypersensitivity pneumonitis (Siersted and Gravesen 1993; Sabino et al. 2019), and “sick-building” syndrome (Dales et al. 1991; Fu et al. 2021) in several studies. In addition, airborne microorganism exposure has been linked to a variety of other health issues, including both the development and the increasing in severity of asthma (Ren et al. 1999). Over 80 genera of fungi have been linked to symptoms of respiratory tract allergies (Horner et al. 1995; Rick et al. 2016; Nageen et al. 2021), and over 100 fungal species are involved in important human and animal infections, while even a greater number of species have been linked to major plant diseases (Cvetnić and Pepeljnjak 1997; Yuan et al. 2022). Numerous studies reported *Cladosporium*, *Alternaria*, *Penicillium*, and *Aspergillus* among the most common fungi in the atmosphere (Shelton et al. 2002; Adhikari et al. 2004; Nageen et al. 2021, 2023; Haas et al. 2023), with varying concentrations from place to place, due to local environmental variables, such as the availability of fungal substrates and the presence of human activities (Shelton et al. 2002). A considerable number of studies on both indoor and outdoor airborne microorganisms have been conducted in different Chinese cities (Wu et al. 2000, 2019; Chen et al. 2010; Liu et al. 2018; Fang et al. 2019; Nageen et al. 2021, 2023; Yuan et al. 2022; Al-Shaarani et al. 2023). These studies have provided useful information about the diversity of airborne fungal communities in the analysed environments, which can be utilised to better understand the implications for human health and to perform risk assessment and disease prevention (Fang et al. 2005, 2019; Nageen et al. 2021, 2023; Lu et al. 2022; Yuan et al. 2022; Nafis et al. 2023). However, little is known about the effect of car traffic on the diversity and structure of airborne fungal communities (Silva et al. 2020). There is still much to learn about the composition and dynamics of fungal communities in urban environments, which are characterised by various indoor and outdoor air conditions, likely to sustain the presence of different fungi (Lee and Jo 2005; Al-Shaarani et al. 2023). Both light and heavy vehicles that consume petroleum fuels have a significant impact on the composition of the atmosphere. Vehicle emissions discharge a variety of harmful air pollutants, such as carbon monoxide and fine particulate matter, which have a tremendous impact on the overall environmental quality (De Toledo and Nardocci 2011; Pérez‐Martínez et al. 2015; Borillo et al. 2018) and may significantly affect human health (Mielnik et al. 2022).

As a large megacity in China, Tianjin has a huge number of vehicles on the roads, which produce major urban pollutants including ozone (O_3_), carbon monoxide (CO), hydrocarbons (HC), nitrogen oxides (NOx), sulphur oxides (SOx), and inhalable particulate matter. Pollution originating from car traffic is well known to be associated with chronic diseases, causing health concerns (André et al. 2000; Habermann et al. 2014; Carvalho et al. 2015; Pérez‐Martínez et al. 2015; Andrade et al. 2017; Mielnik et al. 2022), while it is not yet clear if and how car traffic has also an indirect effect on human health by influencing the distribution and concentration of fungi in the air. In the present study, an airborne fungal community analysis was conducted at high traffic density junctions, in Tianjin, China. We investigated the environmental factors affecting the diversity and concentration of airborne fungi in the selected sites. In particular, we analysed the relationship between fungal community structure, vehicular presence, and meteorological parameters by sampling airborne fungi at different times of the day, characterised by different car traffic intensity during off-peak hours, when car traffic was at its lowest intensity, and peak hours (rush hours), when car traffic reached its highest intensity.

## 2. Materials and methods

### 2.1. Sampling locations

**Figure 1.**
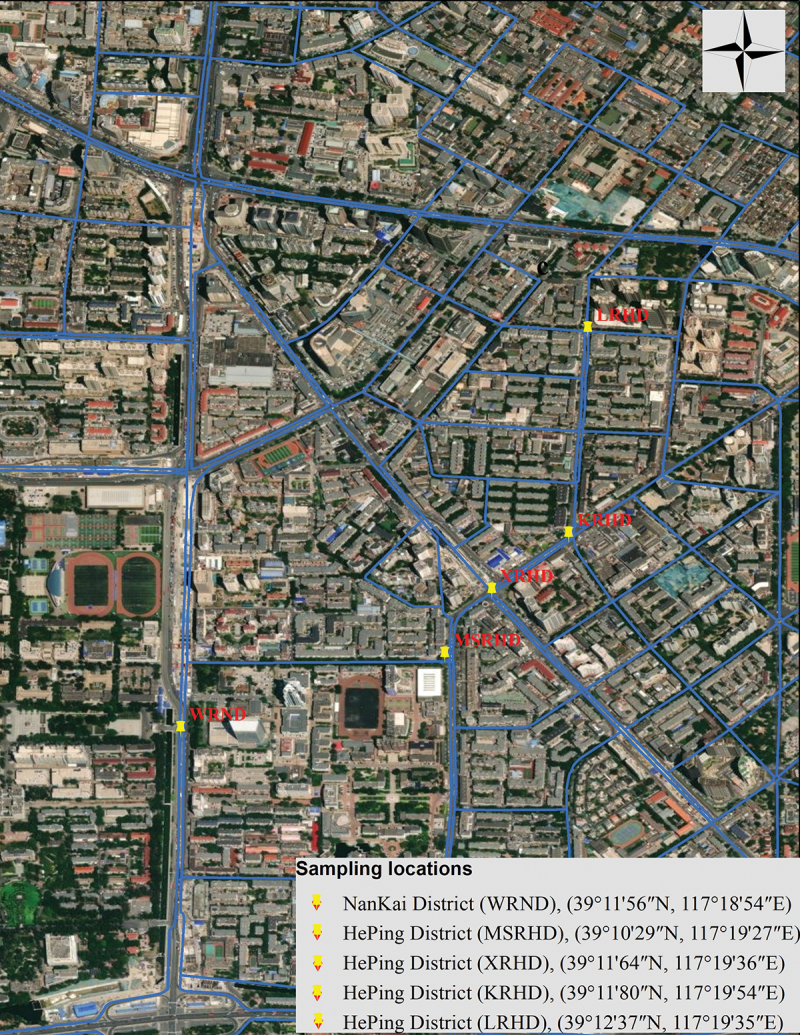
Location of the sampling sites in two districts of Tianjin city, China. The map was generated from ArcGIS 10.8.

This research was carried out in Tianjin, China’s fifth-largest city, located in the northeastern part of China (39°8’31.99“N, 117°10’36.01“E). The city was built in 1403 on the banks of Hai River, West of Bohai Sea, South of Yanshan Mountains, 135 km southeast of Beijing. With 13,794,450 residents in 2021, Tianjin is the fourth most populous city in China, the world’s 29th most populous city, and the 11th most populous urban agglomeration (data retrieved from https://worldpopulationreview.com/world-cities/tianjin-population, accessed on 7 April 2021 and https://www.nationsonline.org/oneworld/map/google_map_Tianjin.htm, accessed on 23 April 2021). Five sampling sites located in different traffic junctions along the main city’s roads were selected for this study (Figure 1). For each site two periods were analysed, including the off-peak period, when the car traffic showed the lowest intensity, and the peak period when the intensity of car traffic was at maximum level (Table S1).

### 2.2. Sampling method

Airborne fungi were sampled, at each study site, once a week, when the weather was dry and stable, for three consecutive weeks, starting from 24 August to 7 September 2021. An air sampler HAS-100b (Hengao T&D, Beijing, China) was employed to collect the culturable airborne fungi from the experimental location using sterile petri dishes of 9 cm in diameter containing Malt Extract Agar (MEA) medium, amended with chloramphenicol (100 mg/L) to prevent bacterial growth. The sampler was placed on a stand at approximately 1.5 m above ground level, which represents the human breathing zone. Air samples were collected at an airflow of 100 L/min, with a rotating dish speed of 0–4 r/min, for 10 min. Before each sampling procedure, to avoid cross-contamination, the air sampler was sterilised by swabbing every surface with cotton dipped in 70% ethanol. Environmental parameters including temperature (T) and relative humidity (RH) were measured during each sampling, using a TES 1364 Humidity-Temperature Metre (Hengao T&D). To understand the influence of car traffic on the analysed airborne fungal communities, two different sampling times were selected, (1) the off-peak period, at 6:30–7:30 am, when there was a very limited presence of cars on the road, and (2) the peak period, at 6:30–7:30 pm, during the rush hour, when the presence of cars reached maximum levels. The collected samples were taken to the lab, where the cultured petri dishes were incubated at room temperature for 5–7 days, in the darkness, and examined every 24 h to detect fungal growth. In each plate, the total number of growing fungal colonies was counted. Each colony was carefully picked and inoculated into a new plate for isolation. The fungal strains collected from the studied sites were identified based on morphological and molecular analyses. All the isolated strains were deposited in the LP Culture Collection (a personal culture collection held in the laboratory of Prof. Lorenzo Pecoraro) at the School of Pharmaceutical Science and Technology, Tianjin University, in Tianjin, China.

### 2.3. Enumeration of fungi

Each week, the number of fungal colonies observed at each sampling location was recorded. The data were transformed into colony-forming units per cubic metre (CFU/m^3^) according to the formula below:

$$N=\frac{\mathrm{Cn}*1,000}{t*V}$$

Where N is the fungal colony concentration in CFU/m^3^, Cn is the number of fungal colonies, 1,000 is the litre to cubic metre conversion factor, t is the sampling operation time, and V is the airflow velocity.

### 2.4. Fungal identification

All the isolated strains were identified based on molecular and morphological analyses. After incubation, fungal mycelium collected from morphologically distinct fungal colonies, isolated from each original sampling plate, was transferred in a 2 mL tube and processed for DNA extraction using the cetyltrimethylammonium bromide technique (Möller et al. 1992; Pecoraro et al. 2012). The following universal primer set was used to amplify the internal transcribed spacer (ITS) region of fungal rRNA: ITS1 (5′-TCCGTAGGTGAACCTGCGG-3′), ITS4 (5′-TCCTCCGCTTATTGATATGC-3′) (White et al. 1990). 25.0 µL of 2× Rapid *Taq* Master Mix (Vazyme Biotech Co. Ltd., Nanjing, China), 2 µL of forward primer (10 µmol/L), 2 µL of reverse primer (10 µmol/L), 2.0 µL of template (20 ng DNA), and 19 µL of double distilled sterilised water were used in the PCR reaction mixture (50 µL). The amplification protocol was as follows: Initial denaturation at 98 °C for 3 min, 30–35 cycles of 98 °C for 10 s, annealing at 55 °C for 15 s, extension at 72 °C for 15 s, and a final extension at 72 °C for 2 min. The PCR products were detected by gel electrophoresis using an electrophoresis tank (LiuYi, Beijing, China) on a 1% agarose gel. The sequencing of PCR products was performed at Genewiz, Inc., Suzhou, China. The sequences were analysed using the National Center for Biotechnology Information’s Basic Local Alignment Search Tool (BLAST) program (http://www.ncbi.nlm.nih.gov/Blast.cgi) to identify the closest matches that enabled taxonomic identification. DNA sequences were deposited in GenBank (Accession No. ON790266–ON790509). Fungal morphology was characterised based on macroscopic and microscopic observations. Microscopy was done using a Nikon ECLIPSE Ci-L microscope (Nikon, Tokyo, Japan) to examine fungal morphological characters, such as hyphae, pseudohyphae, conidiophores, conidia, poroconidia, and arthroconidia, etc (Pecoraro et al. 2021; Wang and Pecoraro 2021).

### 2.5. Statistical analysis

Principal Co-ordinates Analysis (PCoA) and Analysis of Similarity (ANOSIM) with 999 permutations were performed to compare dissimilarities between samples in different locations based on the Bray-Curtis distance (Bray and Curtis 1957) in the R package (version 4.2.2 R Core Team 2022). The differences in fungal genera between car traffic off-peak and peak periods in each site were assessed by the Wilcoxon rank-sum test (Wilcoxon 1945). Between-group Venn Diagrams were plotted to identify unique and common fungal genera (Conway et al. 2017). Distance-based Redundancy Analysis (dbRDA) was performed to relate the environmental variables with the culturable fungal community based on the Bray-Curtis distance matrice, using vegan package (version 2.6-4; Oksanen et al. 2022). The statistical significance of environmental factors was evaluated by Permutational Multivariate Analysis of Variance (PERMANOVA) based on the Bray-Curtis distance. Correlations between fungal genera and environmental parameters were evaluated based on Spearman’s correlation coefficients (*r*) and visualised by Pheatmap (version 1.0.12; Kolde 2019).

## 3. Results

**Figure 2.**
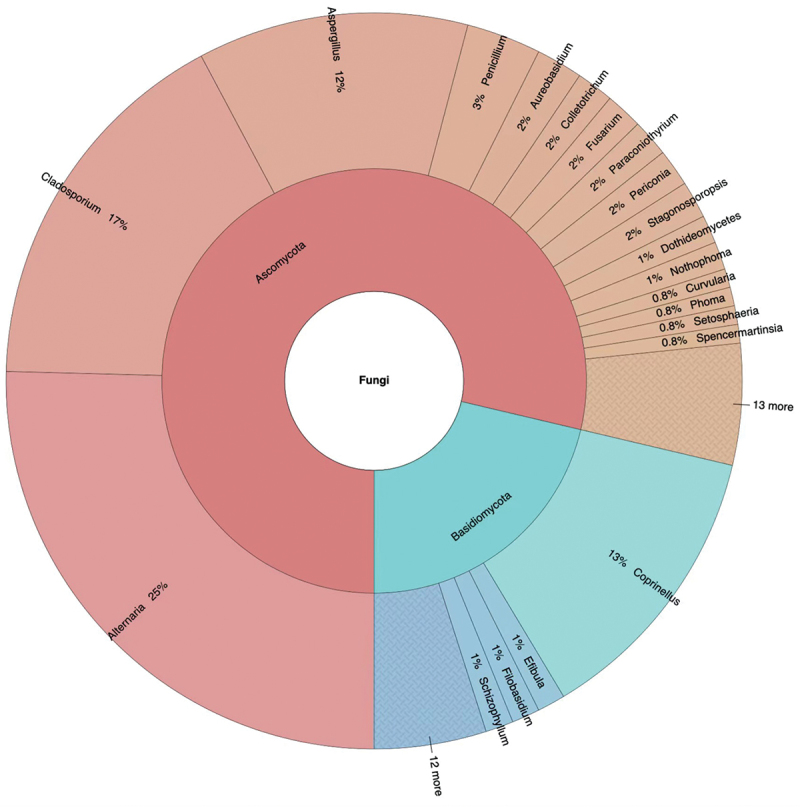
Taxonomic identification and relative abundance of airborne fungi isolated from all selected sites.

A total of 244 fungal strains belonging to 78 species and 45 genera of Ascomycota (78.69%) and Basidiomycota (21.31%) were isolated from the five analysed sites in three consecutive weeks (Figures 2 and S1, Table S2).

*Aspergillus* was the species-richest genus, with 9 species, accounting for 11.54% of the total identified taxa, followed by *Alternaria* and *Cladosporium*, both with 8 species, accounting for 10.3% of the isolated fungal diversity. *Coprinellus radians* was the most abundant fungal species, with 31 strains, yielding 12.70% of the total isolates, followed by *Alternaria alternata* (26 strains, 10.66%), *Cladosporium cladosporioides* (21, 8.6%), *Alternaria compacta* (13, 5.30%), *Cladosporium tenuissimum* (11, 4.50%), and the three species *Alternaria tenuissima*, *Aspergillus versicolor*, and *Penicillium oxalicum*, all showing a 3.28% value of relative abundance.

**Figure 3.**
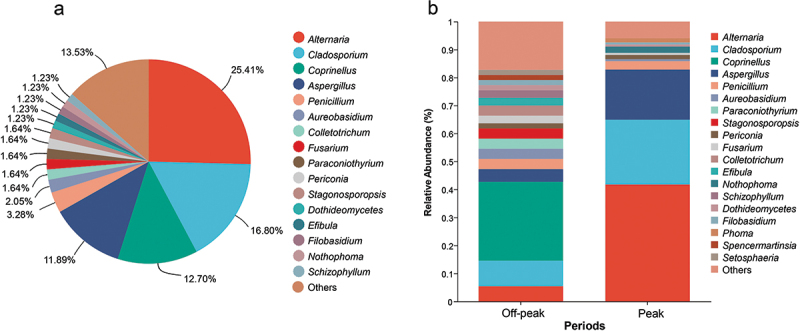
Relative abundance of airborne fungal genera isolated from the analyzed sites. (a) Overview of total fungal diversity from all sampling sites. (b) Genus-level fungal diversity during off-peak and peak periods. Fungal genera with very low relative abundances (<1%) were merged as “others” in the bar plot.

Among the common fungal genera shared by all sampling sites, *Alternaria* accounted for 25.41% of the total isolated strains, followed by *Cladosporium* (16.80%), *Coprinellus* (12.70%), and *Aspergillus* (11.89%; Figure 3a, Table S3). Looking at the different sampling periods (off-peak and peak), *Alternaria* (41.79%), *Cladosporium* (23.13%), and *Aspergillus* (17.91%), showed the highest relative abundances during car traffic peak hours, whereas *Coprinellus* was the most abundant fungal genus found during off-peak periods, accounting for 28.18% of the total isolated strains, followed by *Cladosporium* (9.09%), *Alternaria* (5.45%), and *Aspergillus* (4.55%; Figure 3b). The most abundant fungal genera recorded in the whole study showed a clear variation in their relative abundance between the two investigated periods of car traffic intensity. More specifically, the relative abundance of *Alternaria* varied from 5.55%, during the off-peak period, to 41.79%, during the car traffic peak, *Coprinellus* showed the highest relative abundance (28.18%) at off-peak, while it was not recorded during peak, *Cladosporium* ranged between 9.09% and 23.13% and *Aspergillus* between 4.55% and 17.91% at off-peak and peak, respectively (Figure 3b).

The highest number of strains was collected in the first week (106), with 57 strains isolated from car traffic off-peak and 49 from peak periods, followed by the third week with 77 strains, while the second week yielded the lowest number of fungal isolates (61), with 21 and 40 strains collected at off-peak and peak traffic periods, respectively (Figure S2). Overall, 55% of the fungal strains were collected during peak hours, while 45% were collected during off-peak period.

Looking at the distribution of dominant fungal genera across different sampling weeks, the highest percentage of *Alternaria* strains (46.77%) was recorded in the third sampling week, whereas the highest relative abundance for *Coprinellus* (74.19%) and *Cladosporium* (48.78%) was recorded in the first sampling week. The highest relative numbers of strains for the fungal genera *Aspergillus* (65.52%) and *Penicillium* (62.50%) were isolated in the second and third sampling weeks, respectively.

**Figure 4.**
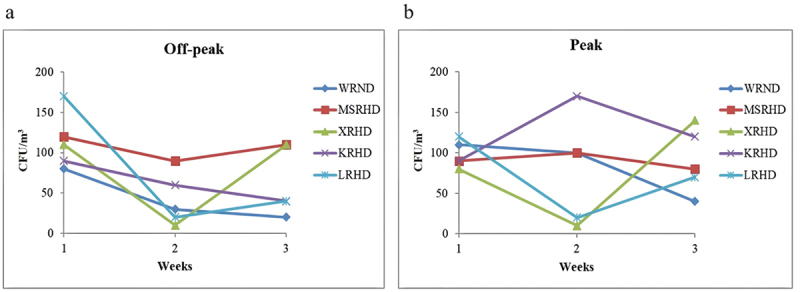
Concentration of airborne fungi during car traffic off-peak (a) and peak (b) in the five analyzed sites during the three-week sampling period (CFU/m^3^). WRND = No. 92, Weijin Road, Nankai District, Tianjin; MSRHD = Meteorological Station Road, Heping District, Tianjin; XRHD = No. 2, Xikang Road, Heping District, Tianjin; KRHD = No. 74 Kunming Road, Heping District, Tianjin; LRHD = Lanzhou Road, Heping District, Tianjin.

The airborne fungal concentrations showed a significant variation across different sites, sampling weeks, and periods of car traffic intensity, ranging from 10 to 170 CFU/m^3^ (Figure 4 and S3, Table S4). In particular, the highest value (170 CFU/m^3^) was recorded at the sampling sites KRHD during car traffic peak and LRHD at off-peak, followed by the level of 140 CFU/m^3^ recorded at the site XRHD during car traffic peak, while the lowest fungal concentration of 10 CFU/m^3^ was recorded at the sampling site XRHD during both off-peak and peak periods. The highest average fungal concentration (126.67 CFU/m^3^) was measured at KRHD (peak), followed by the value of 106.67 CFU/m^3^ recorded at MSRHD (off-peak), whereas the lowest average fungal presence (43.33 CFU/m^3^) was found at WRND during off-peak (Table 1). At off-peak periods, the fungal concentration was found to be constantly higher during week 1 compared to weeks 2 and 3, in all sites except XRHD, where the concentration showed the same value (110 CFU/m^3^) in weeks 1 and 3. The highest off-peak fungal concentration was detected at site LRHD during the first week of sampling. The second week of sampling showed the lowest level of fungal concentration at the site XRHD. The fungal concentration increased at the sites MSRHD, XRHD, and LRHD, while it decreased at the sites WRND and KRHD, during week 3 compared to week 2 (Figure 4a, Table 1). During peak periods, the concentration of airborne fungi at the sites LRHD and WRND during the first week was higher compared to weeks 2 and 3, while it was lower at KRHD. Both the highest and the lowest values of CFU/m^3^ were recorded during the second week at KRHD and XRHD, respectively (Figure 4b).

**Table 1.** Fungal colony concentration in each sampling site.

Sampling site Periods		Mean ‎(CFU/m^3^‎)‎	SD	Min ‎ (CFU/m^3^‎)‎	Max (CFU/m^3^‎)‎
WRND	Off-peak	43.33	32.15	20	80
	Peak	83.33	37.86	40	110
MSRHD	Off-peak	106.67	15.28	90	120
	Peak	90.00	10.00	80	100
XRHD	Off-peak	76.67	57.74	10	110
	Peak	76.67	65.06	10	140
KRHD	Off-peak	63.33	25.17	40	90
	Peak	126.67	40.41	90	170
LRHD	Off-peak	76.67	81.45	20	170
	Peak	70.00	50.00	20	120

Mean = The average fungal concentration; SD = Standard Deviation. WRND = No. 92, Weijin Road, Nankai District, Tianjin; MSRHD = Meteorological Station Road, Heping District, Tianjin; XRHD = No. 2, Xikang Road, Heping District, Tianjin; KRHD = No. 74 Kunming Road, Heping District, Tianjin; LRHD = Lanzhou Road, Heping District, Tianjin.

There was a significant difference in the relative abundance of fungal genera between the off-peak and peak periods (Table S5). In particular, the genera *Alternaria*, *Aspergillus*, and *Cladosporium* were more abundant during the peak of car traffic, while the genus *Coprinellus* was only present in off-peak periods, and the genus *Penicillium* showed an equal presence in both off-peak and peak periods (Table 2, Figure S4).

**Table 2.** Distribution of the 5 most abundant fungal genera during off-peak and peak periods.

Fungal genera	Strains at off-peak	Strains at peak	Total strains
*Alternaria*	6	56	62
*Aspergillus*	5	24	29
*Cladosporium*	10	31	41
*Coprinellus*	31	0	31
*Penicillium*	4	4	8

The Shannon index (H’) values for fungal diversity in different sampling locations for each sampling week are reported in (Table 3). The airborne fungal diversity in off-peak periods showed a peak of 2.10 at the XRHD site in the first week and the lowest value (0) in the second week, while for the peak periods, both the highest (1.70) and the lowest (0) Shannon index values were recorded in the second week, at MSRHD and XRHD, respectively.

**Table 3.** Shannon index of fungal community diversity in different sampling locations, periods of car traffic intensity (off-peak/Peak), and weeks.

Sampling locations	WRND		MSRHD		XRHD		KRHD		LRHD	
	Off-peak	Peak	Off-peak	Peak	Off-peak	Peak	Off-peak	Peak	Off-peak	Peak
First week	1.49	0.92	1.97	0.97	2.10	1.49	1.15	1.43	1.58	0.92
Second week	1.10	0.33	1.83	1.70	0	0	0.69	0.79	0.69	0.69
Third week	0.69	0.69	1.67	0.74	2.02	1.17	1.39	0.29	1.04	1.48

WRND = No. 92, Weijin Road, Nankai District, Tianjin; MSRHD = Meteorological Station Road, Heping District, Tianjin; XRHD = No. 2, Xikang Road, Heping District, Tianjin; KRHD = No. 74 Kunming Road, Heping District, Tianjin; LRHD = Lanzhou Road, Heping District, Tianjin.

Out of the 45 fungal genera detected in the whole study, 9 were exclusively found in the off-peak period, while 26 genera were only detected in the peak period. Only 10 genera were found to be shared by the different airborne fungal communities recorded at off-peak and peak traffic periods (Figure S5).

**Figure 5.**
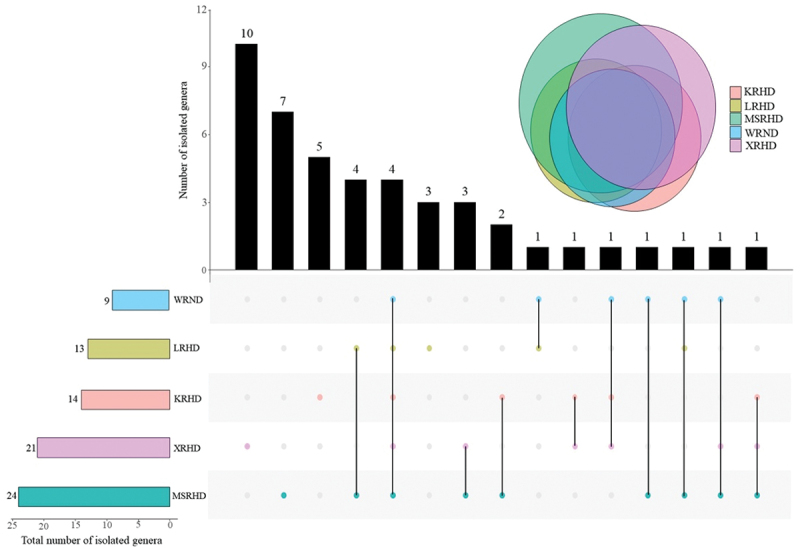
Number of shared and exclusive fungal genera collected from the five sampling sites by upset-Venn diagram. The black columns in the upper part of the graph represent the number of genera present in the sites marked with a colored dot in the lower part of the graph. WRND = No. 92, Weijin Road, Nankai District, Tianjin; MSRHD = Meteorological Station Road, Heping District, Tianjin; XRHD = No. 2, Xikang Road, Heping District, Tianjin; KRHD = No. 74 Kunming Road, Heping District, Tianjin; LRHD = Lanzhou Road, Heping District, Tianjin.

The fungal communities of the five studied sites showed four common genera, namely *Alternaria*, *Aspergillus*, *Cladosporium*, and *Coprinellus*, while *Colletotrichum*, *Penicillium*, *Periconia*, and *Stagonosporopsis* were shared by three out of five sites (Figure 5). The site XRHD showed the highest number of unique genera (10), followed by the sites MSRHD (7), KRHD (5), and LRHD (3). No exclusive genera were recorded in the site WRND (Figure 5). The site MSHRD exhibited the highest total number of genera (24), while the lowest diversity of fungal genera (9) was recorded at WRND (Figure 5).

**Figure 6.**
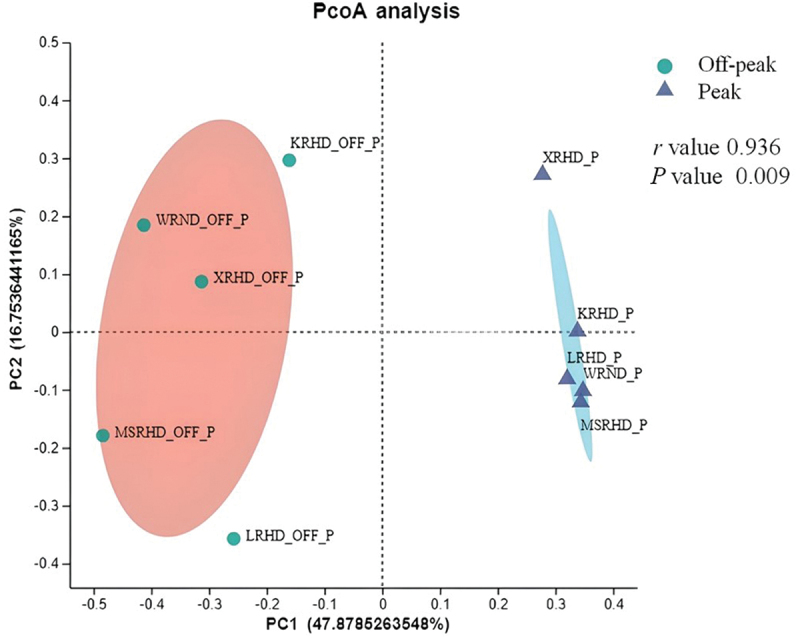
Principal coordinate analysis (PCoA) plots of fungal communities at the car traffic off-peak and peak sampling periods based on Bray-Curtis distance. The *r* and *P* values of the analysis of similarity (ANOSIM) were reported in the figure (*P* < 0.05). WRND = No. 92, Weijin Road, Nankai District, Tianjin; MSRHD = Meteorological Station Road, Heping District, Tianjin; XRHD = No. 2, Xikang Road, Heping District, Tianjin; KRHD = No. 74 Kunming Road, Heping District, Tianjin; LRHD = Lanzhou Road, Heping District, Tianjin.

The variations of fungal genera were analysed by principal co-ordinate analysis (PCoA). The distributions of fungal communities during car traffic off-peak and peak were clearly distinguished based on Bray-Curtis distance (Figure 6). The *r* value was close to 1 (*r* = 0.936), which indicates that the difference between groups is greater than the difference within groups. The *P* value calculated from ANOSIM was 0.009 (*P* < 0.05), suggesting that the differences in fungal communities between the two periods (during off-peak and peak traffic periods) were statistically significant. The Wilcoxon rank-sum test, which was used to investigate the variation of abundant genera among the two analysed periods, showed a difference between car traffic off-peak and peak hours, which was not significant in terms of *P* values. For instance, *Alternaria* and *Coprinellus*, which were the dominant genera during peak and off-peak hours, respectively, showed non-significant differences between the two traffic periods (*P* = 0.2511). An adjusted value of *P* < 0.05 was considered statistically significant (Figure S6).

**Figure 7.**
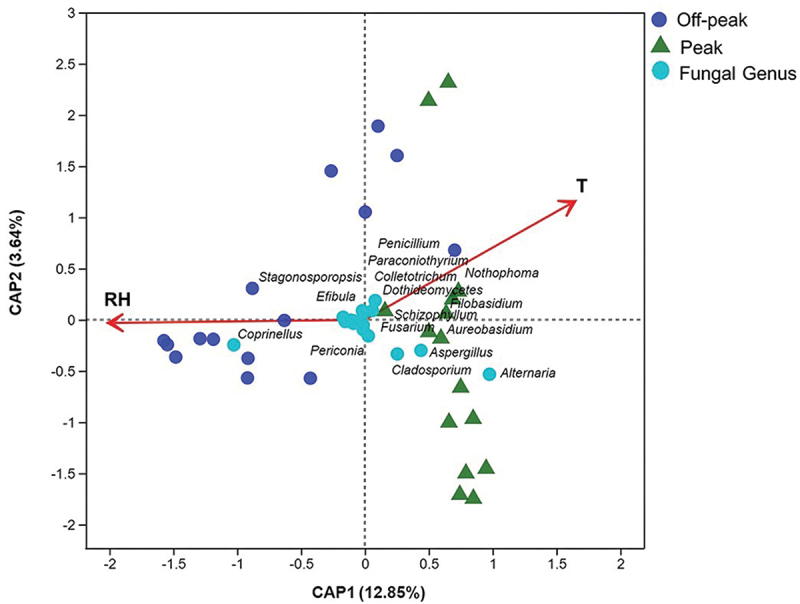
Influence of environmental variables on the fungal community. Distance-based redundancy analysis of the fungal communities, with symbols coded by sampling locations and fungal genera, while T and RH refer to “temperature” and “relative humidity”, respectively.

**Figure 8.**
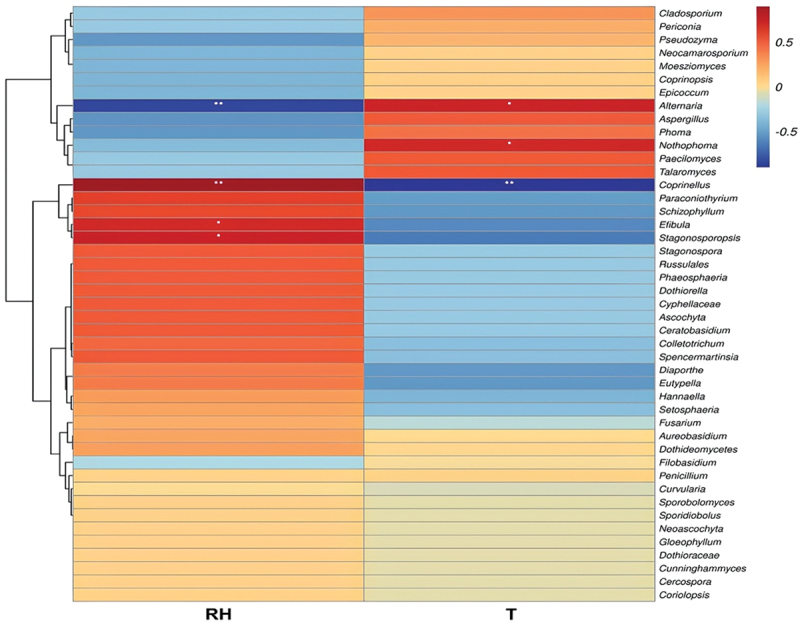
Correlation heatmap between the detected 45 fungal genera and the analyzed environmental factors (RH = Relative humidity, *T* = Temperature). Different colors infer Spearman’s correlation coefficients (*r*). The value *P* is indicated as: *P < 0.05, **P < 0.01.

The dbRDA based on fungal genera showed that both relative humidity and temperature were significantly correlated (*P* = 0.001) with fungal community composition (Figure 7, Tables S6 and S7). The influence of environmental factors on the fungal community varied in the two different investigated periods. For peak hours, temperature showed a higher positive correlation with the fungal communities compared with relative humidity, as revealed by the spots representing fungal communities during peak hours concentrated near the temperature arrow. By contrast, relative humidity showed greater positive impacts on the fungal communities at off-peak hours (Figure 7). For some fungal taxa, such as *Alternaria*, *Aspergillus*, *Cladosporium*, and *Coprinellus*, abundances were much more affected by the analysed environmental factors than for other genera (Figure 7). The Correlation Heatmap showed the influences of environmental variables on all 45 detected fungal genera (Figure 8). Among them, the *Alternaria* genus, which was dominant at peak hours, showed a positive correlation with temperature and a negative correlation with relative humidity. In contrast, the most abundant genus at off-peak hours, *Coprinellus*, exhibited a positive correlation with relative humidity and a negative correlation with temperature. Three fungal genera were significantly correlated with only one of the two recorded factors, with *Nothophoma* showing a positive correlation with temperature, while *Efibula* and *Stagonosporopsis* were positively correlated with relative humidity (Figure 8).

## 4. Discussion

This is the first study on culturable airborne fungi at traffic junctions in Tianjin City, China. The research project was carried out to understand the effect of car traffic on the diversity and structure of airborne fungal communities. The five sites, analysed in three consecutive weeks, during car traffic off-peak and peak periods, yielded a total of 244 strains belonging to 78 species in 45 genera. The isolated fungi belonged to Ascomycota in the majority of cases and, to a much lower extent, to Basidiomycota. In comparison to previous studies conducted in other Chinese cities, such as Beijing and Shenzhen, the airborne fungal communities in Tianjin showed higher diversity. For instance, 40 species belonging to 14 genera were identified in Beijing (Fang et al. 2005), while 27 species in 18 genera were recorded in Shenzhen (Li et al. 2010).

A fungal concentration varying from 10 to 170 CFU/m^3^ was observed during the three consecutive weeks of our research. This result was not surprising, since large variations of airborne fungal concentration have been frequently measured in previous studies, due to the influence of several environmental parameters. For instance, previous works showed that the diversity and concentration of airborne fungi in various outdoor environments were dependent on numerous factors, such as traffic flow, human activities, and the presence of vegetation (di Giorgio et al. 1996; Pasanen et al. 1996; Awad 2002; Nageen et al. 2021, 2023; Lu et al. 2022; Yuan et al. 2022; Al-Shaarani et al. 2023). Varying levels of airborne fungal concentrations were recorded in numerous cities across different countries (di Giorgio et al. 1996; Shelton et al. 2002; Fang et al. 2019; Kalyoncu 2019). For example, in a study conducted in different regions of the United States (Shelton et al. 2002) a median outdoor fungal concentration of 500 CFU/m^3^ was recorded, while Kalyoncu (2019) observed an average concentration of culturable fungi equal to 412 CFU/m^3^ in eight different environments in the city of Yunusemre, Turkey. In China, a study conducted in outdoor environments in Beijing (Fang et al. 2005) reported a fungal concentration range between 24 and 13,960 CFU/m^3^, with the mean and median being 1,165 CFU/m^3^ and 710 CFU/m^3^, respectively. Fang et al. (2008) observed a fungal concentration ranging from 4.8 × 10^2^ to 2.4 × 10^4^ CFU/m^3^ in three different sampling sites in Beijing, whereas the concentration of culturable fungi in the air was between < 12 and 8,767 CFU/m^3^, with a mean of 848 CFU/m^3^, in four sampling locations characterised by different urban functions in Hangzhou (Fang et al. 2019).

The most abundant fungal genera found in Tianjin included *Alternaria*, *Cladosporium*, *Coprinellus*, and *Aspergillus*. Similarly, in previous studies conducted in Nanjing, Beijing, and Hangzhou (Fang et al. 2005, 2019; Wu et al. 2019), *Alternaria* and *Cladosporium* were identified as the most common airborne fungi. *Alternaria* was the dominant genus recorded in our study, comprising almost 25.40% of all the airborne fungal strains that were isolated over three weeks of analysis. Among the recorded *Alternaria* species, *A. alternata* was the most abundant, accounting for 10.66% of all isolated strains. *Alternaria alternata* is widely recognised as one of the most frequent airborne fungal species in outdoor and indoor environments. This fungus is known to be an aeroallergen and a source of allergic reactions in sensitive patients (Hedayati et al. 2009). Moreover, *A. alternata* was found to be responsible for immunoglobulin E (IgE)-mediated respiratory illnesses, particularly asthma exacerbation (Downs et al. 2001). A total of 16 allergens associated with *A. alternata* have been identified (Kustrzeba-Wójcicka et al. 2014; Gabriel et al. 2016). *Cladosporium* was the second most common genus detected in Tianjin air environments, while *C. cladosporioides* was the third most abundant fungal species, accounting for 8.6% of the total number of strains. Although *Cladosporium* species are mainly saprotrophic (Larsen 1981), they have been recognised as opportunistic pathogens in animals and humans (Mercier et al. 2013; Sandoval-Denis et al. 2015). *Cladosporium* was reported as the prevalent fungal genus in nasal microbiota, with *C. herbarum* and *C. cladosporioides* being the most abundant species, in a study performed by Sellart-Altisent et al. (2007). In a clinical study carried out in China, *C. cladosporioides* was reported as a cause of phaeohyphomycotic dermatitis in giant pandas (Ma et al. 2013).

Numerous aeromycological studies have been published on the effect of meteorological factors on airborne fungal concentration (Picco and Rodolfi 2000; Giri et al. 2008; Kim et al. 2009; Ponce-Caballero et al. 2013; Hwang and Cho 2016; Hwang et al. 2016; Nageen et al. 2021; Lu et al. 2022; Al-Shaarani et al. 2023). Previous research has shown that environmental parameters such as temperature, humidity, wind speed, and atmospheric pressure affect the diversity of airborne fungi (Larsen 1981; Yuan et al. 2022; Haas et al. 2023; Nageen et al. 2023). The analysis performed in our work revealed a significant effect of temperature and relative humidity on the diversity and concentration of airborne fungi present in the investigated urban areas. These results are consistent with the findings of previous studies that reported a major influence of temperature and humidity on airborne fungal communities. For example, in a study conducted in Hangzhou by Fang et al. (2019), the air temperature was found to sustain fungal growth and germination in all seasons except winter, whereas a work carried out in Beijing showed that both air temperature and humidity were more appropriate for fungal propagation in summer and autumn than in winter, thus significantly affecting the analysed airborne fungal communities (Fang et al. 2005).

Concerning the effect of car traffic on microbial air quality, our results showed dramatic differences in the diversity and concentration of airborne fungi between the two analysed periods characterised by different car traffic intensities (off-peak and peak). We found a relationship between the number of vehicles on the road and the presence of viable fungal spores in urban air environments, resulting in greater fungal diversity during peak traffic hours, particularly with a higher concentration of dominant fungal genera (*Alternaria*, *Aspergillus*, and *Cladosporium*) compared to off-peak times. The positive correlation between the intensity of car traffic and the diversity of airborne fungi in the analysed city environments could be due to fuel emissions and increasing pollution in the air. Further studies are needed to test this hypothesis and to describe in detail the variation of air parameters related to the presence of car traffic, which in turn may affect the structure of the airborne fungal communities. Different results were found in a previous study conducted in São Paulo, Brazil, where the number of isolated airborne fungal colonies was related to atmospheric conditions and the presence of air pollutants (Silva et al. 2020). In the latter work, the number of colony-forming units for the fungal genera *Aspergillus*, *Curvularia*, *Penicillium*, *Neurospora*, *Rhizopus*, and *Trichoderma* increased by approximately 80% during the sampling period, in response to environmental changes favoured by the reduction of vehicle presence (Silva et al. 2020). Several environmental factors related to the differences in climate and geography could be responsible for the contrasting results between our study, conducted in a temperate environment, and the study conducted in Brazil, in a tropical climate. Besides, different levels of pollution and air quality in the two cities could impact the growth and distribution of fungi. For instance, different types of vehicles and fuel emissions may result in different types and amounts of pollutants released in the air of the two cities, which may have varying effects on airborne fungal communities. Future studies based on a large number of environmental parameters either related to climate conditions or human activity and air pollution will be essential to better understand the effects of car traffic on airborne fungal diversity and concentration. The use of a metabarcoding approach could be important in revealing the total diversity of airborne fungal communities in urban environments by detecting the presence of unculturable fungi that might affect human health.

## 5. Conclusions

The current population growth in major cities around the world has a series of harmful consequences, especially in terms of increasing air pollution. Due to the obvious direct correlation between air quality and human health, it is of critical concern to investigate the diversity and concentration of microbes present in the air in urban areas. Airborne fungi can be monitored to detect pollution-related atmospheric conditions. In this study, we shed light on the diverse community of airborne fungi that characterised the investigated outdoor environments at Tianjin traffic junctions. The diversity and concentration of airborne fungi varied with changes in environmental conditions. We confirmed our hypothesis of a relationship between car traffic and the presence of viable fungal propagules in urban air. This led to increased diversity and concentration of airborne fungi during peak hours when car traffic was at its highest intensity. Higher intensity of car traffic resulted in higher concentrations of fungal particles in the air for various taxa, including *Alternaria*, *Aspergillus*, and *Cladosporium*, which are known to cause respiratory allergies and infections. This result suggests that reducing vehicular traffic or implementing measures to control dust and emissions could be effective measures to control airborne fungal exposure and microbial pollution. This study showed how air quality in urban areas might affect the structure of airborne fungal communities and, therefore, the health of people. Overall, the study highlights the importance of understanding the complex interplay between environmental factors and fungal exposure to assess human health risks, and the need for targeted interventions to improve outdoor air quality. Understanding the dynamics of fungal communities under different environmental conditions is crucial to planning strategies for mitigating fungal exposure in outdoor environments. The results of our research could be taken into consideration to guide government policies and may represent a valuable source of information for the prevention of airborne-related diseases in urban areas.

## Supplementary Material

Supplemental Material

## Data Availability

Data presented in this study can be found as part of the manuscript, and in the Supplementary Materials. The fungal DNA sequences amplified during this study are available at GenBank under accessions ON790266–ON790509.
