# Supplementary material for: The influence of car traffic on airborne fungal diversity in Tianjin, China
Source: Mycology. 2024 Jan 18;15(3):506–20. doi: 10.1080/21501203.2023.2300343 (PMC11376297; doi:10.1080/21501203.2023.2300343)
Supplement: Supplemental Material [file TMYC_A_2300343_SM1926.docx]

**The influence of car traffic on airborne fungal diversity in Tianjin, China.**

**Mohammed H.M. Muafa^1^, Ziwei M. Quach^1^, Amran A.Q.A. Al-Shaarani^1^, Md M.H. Nafis^1^, Lorenzo Pecoraro^1^***

^1^School of Pharmaceutical Science and Technology, Tianjin University, 92 Weijin Road, Tianjin 300072, China.

*Correspondence: Lorenzo Pecoraro (telephone: +86 18520824550, e-mail: lorenzo.pecoraro@tju.edu.cn) School of Pharmaceutical Science and Technology, Tianjin University, 92 Weijin Road, Nankai District, Tianjin 300072, China.

**Supplementary information**

**Supplementary Table S1.** Details of the selected sampling sites.‎

| **No. 92, Weijin Road, Nankai District, Tianjin (WRND)** | |
| --- | --- |
| **Sampling site** | **GPS** |
| Off-peak | 39°11'56″N 117°18'54″E |
| Peak | 39°11'56″N 117°18'54″E |
| **Meteorological Station Road, Heping District, Tianjin (MSRHD)** | |
| **Sampling site** | **GPS** |
| Off-peak | 39°10'29″N 117°19'27″E |
| Peak | 39°10'29″N 117°19'27″E |
| **No. 2, Xikang Road, Heping District, Tianjin (XRHD)** | |
| **Sampling site** | **GPS** |
| Off-peak | 39°11'64″N 117°19'36″E |
| Peak | 39°11'64″N 117°19'36″E |
| **No. 74 Kunming Road, Heping District, Tianjin (KRHD)** | |
| **Sampling site** | **GPS** |
| Off-peak | 39°11'80″N 117°19'54″E |
| Peak | 39°11'80″N 117°19'54″E |
| **Lanzhou Road, Heping District, Tianjin (LRHD)** | |
| **Sampling site** | **GPS** |
| Off-peak | 39°12'37″N 117°19'35″E |
| Peak | 39°12'37″N 117°19'35″E |

**Supplementary Table S2.** Airborne fungal diversity molecularly detected in Tianjin outdoor environments, from DNA extracted from isolated strains.

BLAST search closest matches of fungal internal transcribed spacer DNA sequences amplified from air samples. Strain GenBank accession codes, accession codes for the closest GenBank matches, sequence identity, and overlap of each match are reported. WRND = No. 92, Weijin Road, Nankai District, Tianjin, MSRHD = Meteorological Station Road, Heping District, Tianjin, XRHD = No. 2, Xikang Road, Heping District, Tianjin, KRHD = No. 74 Kunming Road, Heping District, Tianjin, LRHD = Lanzhou Road, Heping District, Tianjin.

| **Sample**  **No.** | **Sampling**  **week** | **Site**  **code** | **GenBank**  **code** | **Best BLAST match(es)** | **Accession**  **code** | **Overlap**  **length** | **%**  **match** |
| --- | --- | --- | --- | --- | --- | --- | --- |
| 1 | First week | WRND Off-peak | ON790266 | *Cladosporium tenuissimum* | [KP689183.1](https://www.ncbi.nlm.nih.gov/nucleotide/KP689183.1?report=genbank&log$=nucltop&blast_rank=1&RID=ZHJ0XDSJ016) | 522 | 99.81% |
| 2 | First week | WRND Off-peak | ON790267 | *Coprinellus radians* | [MK087751.1](https://www.ncbi.nlm.nih.gov/nucleotide/MK087751.1?report=genbank&log$=nucltop&blast_rank=1&RID=5NZXK83P013) | 729 | 99.54% |
| 3 | First week | WRND Off-peak | ON790268 | *Filobasidium magnum* | [OM236818.1](https://www.ncbi.nlm.nih.gov/nucleotide/OM236818.1?report=genbank&log$=nucltop&blast_rank=1&RID=ZHJV3RSC013) | 592 | 96.09% |
| 4 | First week | WRND Off-peak | ON790269 | *Coprinellus radians* | [MK732139.1](https://www.ncbi.nlm.nih.gov/nucleotide/MK732139.1?report=genbank&log$=nucltop&blast_rank=4&RID=5NZ5SJMA013) | 672 | 99.69% |
| 5 | First week | WRND Off-peak | ON790270 | *Coprinellus radians* | [KP900252.1](https://www.ncbi.nlm.nih.gov/nucleotide/KP900252.1?report=genbank&log$=nucltop&blast_rank=3&RID=5P0P3BHS013) | 658 | 99.24% |
| 6 | First week | WRND Off-peak | ON790271 | *Colletotrichum gloeosporioides* | [MN833335.1](https://www.ncbi.nlm.nih.gov/nucleotide/MN833335.1?report=genbank&log$=nucltop&blast_rank=1&RID=ZM5ZM06U01R) | 580 | 99.45% |
| 7 | First week | WRND Off-peak | ON790272 | *Stagonosporopsis* sp.  *Stagonosporopsis caricae* | [MN871629.1](https://www.ncbi.nlm.nih.gov/nucleotide/MN871629.1?report=genbank&log$=nucltop&blast_rank=1&RID=ZM6G4G0F013)  [MH863092.1](https://www.ncbi.nlm.nih.gov/nucleotide/MH863092.1?report=genbank&log$=nucltop&blast_rank=2&RID=ZM6G4G0F013) | 521  572 | 98.53%  98.53% |
| 8 | First week | WRND Off-peak | ON790273 | *Colletotrichum* sp. | [MN856366.1](https://www.ncbi.nlm.nih.gov/nucleotide/MN856366.1?report=genbank&log$=nucltop&blast_rank=1&RID=ZMHYPYV4016) | 583 | 99.81% |
| 9 | First week | MSRHD Off-peak | ON790274 | *Cladosporium cladosporioides* | [KX258800.1](https://www.ncbi.nlm.nih.gov/nucleotide/KX258800.1?report=genbank&log$=nucltop&blast_rank=2&RID=8210XKBP016) | 526 | 96.14% |
| 10 | First week | MSRHD Off-peak | ON790275 | *Cladosporium oxysporum* | [OM236950.1](https://www.ncbi.nlm.nih.gov/nucleotide/OM236950.1?report=genbank&log$=nucltop&blast_rank=1&RID=ZMK58EJ9016) | 535 | 99.81% |
| 11 | First week | MSRHD Off-peak | ON790276 | *Coprinellus radians* | [MK087751.1](https://www.ncbi.nlm.nih.gov/nucleotide/MK087751.1?report=genbank&log$=nucltop&blast_rank=1&RID=ZMKH3WBM016) | 729 | 99.54% |
| 12 | First week | MSRHD Off-peak | ON790277 | *Stagonosporopsis cucurbitacearum* | [LC168795.1](https://www.ncbi.nlm.nih.gov/nucleotide/LC168795.1?report=genbank&log$=nucltop&blast_rank=1&RID=ZMM4YNF4016) | 531 | 99.42% |
| 13 | First week | MSRHD Off-peak | ON790278 | *Cyphellaceae* sp. | [MG269994.1](https://www.ncbi.nlm.nih.gov/nucleotide/MG269994.1?report=genbank&log$=nucltop&blast_rank=1&RID=ZMMKS8K1013) | 702 | 99.40% |
| 14 | First week | MSRHD Off-peak | ON790279 | *Colletotrichum aenigma* | [MT476807.1](https://www.ncbi.nlm.nih.gov/nucleotide/MT476807.1?report=genbank&log$=nucltop&blast_rank=1&RID=ZMMZDTNM01R) | 618 | 99.44% |
| 15 | First week | MSRHD Off-peak | ON790280 | *Coprinellus radians* | [MT898685.1](https://www.ncbi.nlm.nih.gov/nucleotide/MT898685.1?report=genbank&log$=nucltop&blast_rank=1&RID=ZMNA8KJ1016) | 678 | 99.24% |
| 16 | First week | MSRHD Off-peak | ON790281 | *Efibula matsuensis* | [NR175181.1](https://www.ncbi.nlm.nih.gov/nucleotide/NR_175181.1?report=genbank&log$=nucltop&blast_rank=1&RID=5U6HNZFD016) | 627 | 99.50% |
| 17 | First week | MSRHD Off-peak | ON790282 | *Coprinellus radians* | [MK087751.1](https://www.ncbi.nlm.nih.gov/nucleotide/MK087751.1?report=genbank&log$=nucltop&blast_rank=1&RID=ZN04F61E013) | 729 | 99.54% |
| 18 | First week | MSRHD Off-peak | ON790283 | *Phaeosphaeria* sp.  *Phaeosphaeria fuckelii* | [HQ914835.1](https://www.ncbi.nlm.nih.gov/nucleotide/HQ914835.1?report=genbank&log$=nucltop&blast_rank=1&RID=ZN0FEZPT016)  [MZ396933.1](https://www.ncbi.nlm.nih.gov/nucleotide/MZ396933.1?report=genbank&log$=nucltop&blast_rank=2&RID=ZN0FEZPT016) | 547  563 | 99.43%  99.24% |
| 19 | First week | MSRHD Off-peak | ON790284 | *Stagonosporopsis cucurbitacearum* | [LC168795.1](https://www.ncbi.nlm.nih.gov/nucleotide/LC168795.1?report=genbank&log$=nucltop&blast_rank=1&RID=ZN0V6492016) | 531 | 99.61% |
| 20 | First week | MSRHD Off-peak | ON790285 | *Coprinellus radians* | [MK732139.1](https://www.ncbi.nlm.nih.gov/nucleotide/MK732139.1?report=genbank&log$=nucltop&blast_rank=1&RID=ZN1YXVD6016) | 672 | 100.00% |
| 21 | First week | XRHD Off-peak | ON790286 | *Cladosporium tenuissimum* | [OM237136.1](https://www.ncbi.nlm.nih.gov/nucleotide/OM237136.1?report=genbank&log$=nucltop&blast_rank=1&RID=ZN2DTA0W016) | 532 | 100.00% |
| 22 | First week | XRHD Off-peak | ON790287 | *Sporidiobolus pararoseus* | [MW081279.1](https://www.ncbi.nlm.nih.gov/nucleotide/MW081279.1?report=genbank&log$=nucltop&blast_rank=1&RID=ZN3DCGXU016) | 608 | 99.47% |
| 23 | First week | XRHD Off-peak | ON790288 | *Sporobolomyces carnicolor* | [KY962981.1](https://www.ncbi.nlm.nih.gov/nucleotide/KY962981.1?report=genbank&log$=nucltop&blast_rank=1&RID=ZN470NW8016) | 603 | 99.29% |
| 24 | First week | XRHD Off-peak | ON790289 | *Coprinellus radians* | [MK087751.1](https://www.ncbi.nlm.nih.gov/nucleotide/MK087751.1?report=genbank&log$=nucltop&blast_rank=3&RID=5XJ7YKSK013) | 729 | 99.38% |
| 25 | First week | XRHD Off-peak | ON790290 | *Cunninghammyces* sp. | [MW557941.1](https://www.ncbi.nlm.nih.gov/nucleotide/MW557941.1?report=genbank&log$=nucltop&blast_rank=1&RID=1F5EU082016) | 702 | 98.68% |
| 26 | First week | XRHD Off-peak | ON790291 | *Dothideomycetes* sp. | [KX908683.1](https://www.ncbi.nlm.nih.gov/nucleotide/KX908683.1?report=genbank&log$=nucltop&blast_rank=1&RID=ZPVGB1TP013) | 1066 | 98.58% |
| 27 | First week | XRHD Off-peak | ON790292 | *Coprinellus radians* | [MK087751.1](https://www.ncbi.nlm.nih.gov/nucleotide/MK087751.1?report=genbank&log$=nucltop&blast_rank=1&RID=ZPW360KP016) | 729 | 99.38% |
| 28 | First week | XRHD Off-peak | ON790293 | *Coprinellus radians* | [MK087751.1](https://www.ncbi.nlm.nih.gov/nucleotide/MK087751.1?report=genbank&log$=nucltop&blast_rank=1&RID=ZPWH2TUE013) | 729 | 99.69% |
| 29 | First week | XRHD Off-peak | ON790294 | *Colletotrichum gloeosporioides* | [MN856359.1](https://www.ncbi.nlm.nih.gov/nucleotide/MN856359.1?report=genbank&log$=nucltop&blast_rank=1&RID=ZPWYP9NJ016) | 582 | 99.63% |
| 30 | First week | XRHD Off-peak | ON790295 | *Neoascochyta* sp. | [MW670506.1](https://www.ncbi.nlm.nih.gov/nucleotide/MW670506.1?report=genbank&log$=nucltop&blast_rank=1&RID=ZPXB37C5013) | 484 | 100.00% |
| 31 | First week | XRHD Off-peak | ON790296 | *Dothioraceae* sp. | [KU892278.1](https://www.ncbi.nlm.nih.gov/nucleotide/KU892278.1?report=genbank&log$=nucltop&blast_rank=1&RID=ZPY3AYDH016) | 564 | 97.94% |
| 32 | First week | KRHD Off-peak | ON790297 | *Coprinellus radians* | [KP900252.1](https://www.ncbi.nlm.nih.gov/nucleotide/KP900252.1?report=genbank&log$=nucltop&blast_rank=4&RID=5XHVVRXW016) | 658 | 99.69% |
| 33 | First week | KRHD Off-peak | ON790298 | *Schizophyllum commune* | [KU042974.1](https://www.ncbi.nlm.nih.gov/nucleotide/KU042974.1?report=genbank&log$=nucltop&blast_rank=1&RID=ZPZ6NNVM016) | 614 | 99.34% |
| 34 | First week | KRHD Off-peak | ON790299 | *Coprinellus radians* | [LC612525.1](https://www.ncbi.nlm.nih.gov/nucleotide/LC612525.1?report=genbank&log$=nucltop&blast_rank=1&RID=ZR157ZFX013) | 1624 | 99.39% |
| 35 | First week | KRHD Off-peak | ON790300 | *Coprinellus radians* | [KP900252.1](https://www.ncbi.nlm.nih.gov/nucleotide/KP900252.1?report=genbank&log$=nucltop&blast_rank=4&RID=5XHA27V7013) | 658 | 91.98% |
| 36 | First week | KRHD Off-peak | ON790301 | *Coprinellus radians* | [MK087751.1](https://www.ncbi.nlm.nih.gov/nucleotide/MK087751.1?report=genbank&log$=nucltop&blast_rank=1&RID=ZR7BVKM7013) | 729 | 99.39% |
| 37 | First week | KRHD Off-peak | ON790302 | *Alternaria tamaracks* | [OM237300.1](https://www.ncbi.nlm.nih.gov/nucleotide/OM237300.1?report=genbank&log$=nucltop&blast_rank=1&RID=ZR85UMM701R) | 550 | 99.44% |
| 38 | First week | KRHD Off-peak | ON790303 | *Bullera* sp.  *Hannaella oryzae* | [MF662376.1](https://www.ncbi.nlm.nih.gov/nucleotide/MF662376.1?report=genbank&log$=nucltop&blast_rank=1&RID=ZR9XNHGG016)  [KY103504.1](https://www.ncbi.nlm.nih.gov/nucleotide/KY103504.1?report=genbank&log$=nucltop&blast_rank=2&RID=ZR9XNHGG016) | 447  646 | 99.33%  99.33% |
| 39 | First week | KRHD Off-peak | ON790304 | *Alternaria angustiovoidea* | [OM237284.1](https://www.ncbi.nlm.nih.gov/nucleotide/OM237284.1?report=genbank&log$=nucltop&blast_rank=1&RID=ZRBACVZ201R) | 555 | 99.63% |
| 40 | First week | KRHD Off-peak | ON790305 | *Coprinellus radians* | [KP900252.1](https://www.ncbi.nlm.nih.gov/nucleotide/KP900252.1?report=genbank&log$=nucltop&blast_rank=1&RID=ZTEYF0Y301R) | 658 | 99.39% |
| 41 | First week | LRHD Off-peak | ON790306 | *Cladosporium cladosporioides* | [OM237138.1](https://www.ncbi.nlm.nih.gov/nucleotide/OM237138.1?report=genbank&log$=nucltop&blast_rank=1&RID=ZTFBUNGW013) | 526 | 100.00% |
| 42 | First week | LRHD Off-peak | ON790307 | *Cladosporium cladosporioides* | [OM237122.1](https://www.ncbi.nlm.nih.gov/nucleotide/OM237122.1?report=genbank&log$=nucltop&blast_rank=1&RID=ZTG15YYZ016) | 533 | 100.00% |
| 43 | First week | LRHD Off-peak | ON790308 | *Fusarium solani* | [KU360147.1](https://www.ncbi.nlm.nih.gov/nucleotide/KU360147.1?report=genbank&log$=nucltop&blast_rank=1&RID=ZU4MYGRA013) | 543 | 91.82% |
| 44 | First week | LRHD Off-peak | ON790309 | *Aspergillus* sp.  *Aspergillus creber* | [MT028069.1](https://www.ncbi.nlm.nih.gov/nucleotide/MT028069.1?report=genbank&log$=nucltop&blast_rank=1&RID=ZU4ZS14E016)  LN898693.1 | 568  760 | 99.81%  99.63% |
| 45 | First week | LRHD Off-peak | ON790310 | *Coprinellus radians* | [MT898685.1](https://www.ncbi.nlm.nih.gov/nucleotide/MT898685.1?report=genbank&log$=nucltop&blast_rank=1&RID=ZU5BK61R01R) | 678 | 99.54% |
| 46 | First week | LRHD Off-peak | ON790311 | *Coprinellus radians* | [MT898685.1](https://www.ncbi.nlm.nih.gov/nucleotide/MT898685.1?report=genbank&log$=nucltop&blast_rank=1&RID=ZU5MH9M001R) | 678 | 99.24% |
| 47 | First week | LRHD Off-peak | ON790312 | *Coprinellus radians* | [MT898685.1](https://www.ncbi.nlm.nih.gov/nucleotide/MT898685.1?report=genbank&log$=nucltop&blast_rank=1&RID=ZU6550J0013) | 678 | 99.24% |
| 48 | First week | LRHD Off-peak | ON790313 | *Eutypella citricola* | [MW081302.1](https://www.ncbi.nlm.nih.gov/nucleotide/MW081302.1?report=genbank&log$=nucltop&blast_rank=1&RID=ZU6FAK6F013) | 588 | 98.54% |
| 49 | First week | LRHD Off-peak | ON790314 | *Coprinellus radians* | [FJ582637.1](https://www.ncbi.nlm.nih.gov/nucleotide/FJ582637.1?report=genbank&log$=nucltop&blast_rank=1&RID=ZU6XMB5W016) | 710 | 99.69% |
| 50 | First week | LRHD Off-peak | ON790315 | *Stagonosporopsis cucurbitacearum* | [LC168795.1](https://www.ncbi.nlm.nih.gov/nucleotide/LC168795.1?report=genbank&log$=nucltop&blast_rank=1&RID=ZU7552B1013) | 531 | 99.80% |
| 51 | First week | LRHD Off-peak | ON790316 | *Fusarium solani* | [JX241656.1](https://www.ncbi.nlm.nih.gov/nucleotide/JX241656.1?report=genbank&log$=nucltop&blast_rank=3&RID=ZU7G3GZJ01R) | 576 | 99.44% |
| 52 | First week | LRHD Off-peak | ON790351 | *Fusarium graminearum* | [KY272777.1](https://www.ncbi.nlm.nih.gov/nucleotide/KY272777.1?report=genbank&log$=nucltop&blast_rank=1&RID=04RZ0P1Z01R) | 515 | 99.41% |
| 53 | First week | LRHD Off-peak | ON790352 | *Coprinellus radians* | [FJ582637.1](https://www.ncbi.nlm.nih.gov/nucleotide/FJ582637.1?report=genbank&log$=nucltop&blast_rank=1&RID=04SAYXSC016) | 710 | 99.54% |
| 54 | First week | LRHD Off-peak | ON790353 | *Coprinellus radians* | [MK087751.1](https://www.ncbi.nlm.nih.gov/nucleotide/MK087751.1?report=genbank&log$=nucltop&blast_rank=1&RID=04TKTV3R013) | 729 | 99.24% |
| 55 | First week | LRHD Off-peak | ON790354 | *Diaporthe caulivora* | [MK828222.1](https://www.ncbi.nlm.nih.gov/nucleotide/MK828222.1?report=genbank&log$=nucltop&blast_rank=1&RID=04TY57V4016) | 605 | 99.45% |
| 56 | First week | LRHD Off-peak | ON790355 | *Coprinellus radians* | [KJ780763.1](https://www.ncbi.nlm.nih.gov/nucleotide/KJ780763.1?report=genbank&log$=nucltop&blast_rank=2&RID=5XFJUHTP013) | 691 | 99.09% |
| 57 | First week | LRHD Off-peak | ON790356 | *Coprinellus radians* | [MT898685.1](https://www.ncbi.nlm.nih.gov/nucleotide/MT898685.1?report=genbank&log$=nucltop&blast_rank=1&RID=04W3WRUK01R) | 678 | 99.39% |
| 58 | First week | WRND Peak | ON790317 | *Aspergillus japonicus* | [KY199566.1](https://www.ncbi.nlm.nih.gov/nucleotide/KY199566.1?report=genbank&log$=nucltop&blast_rank=1&RID=ZU88UY0G013) | 683 | 99.26% |
| 59 | First week | WRND Peak | ON790318 | *Aspergillus japonicus* | [MT528855.1](https://www.ncbi.nlm.nih.gov/nucleotide/MT528855.1?report=genbank&log$=nucltop&blast_rank=1&RID=ZU8FV3RM013) | 564 | 99.63% |
| 60 | First week | WRND Peak | ON790319 | *Alternaria angustiovoidea* | [MW009022.1](https://www.ncbi.nlm.nih.gov/nucleotide/MW009022.1?report=genbank&log$=nucltop&blast_rank=2&RID=ZU8ZN9ET01R) | 551 | 99.27% |
| 61 | First week | WRND Peak | ON790320 | *Aspergillus aculeatus* | [MN795742.1](https://www.ncbi.nlm.nih.gov/nucleotide/MN795742.1?report=genbank&log$=nucltop&blast_rank=2&RID=5XGM6V77013) | 589 | 99.45% |
| 62 | First week | WRND Peak | ON790321 | *Alternaria compacta* | [OM236750.1](https://www.ncbi.nlm.nih.gov/nucleotide/OM236750.1?report=genbank&log$=nucltop&blast_rank=1&RID=ZU9WSZAZ013) | 555 | 99.45% |
| 63 | First week | WRND Peak | ON790322 | *Alternaria alternata* | [OM237005.1](https://www.ncbi.nlm.nih.gov/nucleotide/OM237005.1?report=genbank&log$=nucltop&blast_rank=1&RID=ZUA9CGYT013) | 502 | 93.78% |
| 64 | First week | WRND Peak | ON790357 | *Aspergillus aculeatus* | [KX865278.1](https://www.ncbi.nlm.nih.gov/nucleotide/KX865278.1?report=genbank&log$=nucltop&blast_rank=2&RID=5XF37218016) | 553 | 99.45% |
| 65 | First week | WRND Peak | ON790358 | *Alternaria alternata* | [OM236773.1](https://www.ncbi.nlm.nih.gov/nucleotide/OM236773.1?report=genbank&log$=nucltop&blast_rank=1&RID=04XN91KC013) | 544 | 97.03% |
| 66 | First week | WRND Peak | ON790359 | *Alternaria angustiovoidea* | [MW009022.1](https://www.ncbi.nlm.nih.gov/nucleotide/MW009022.1?report=genbank&log$=nucltop&blast_rank=1&RID=04Z6C563016) | 551 | 99.81% |
| 67 | First week | WRND Peak | ON790360 | *Alternaria tamaricis* | [OM236768.1](https://www.ncbi.nlm.nih.gov/nucleotide/OM236768.1?report=genbank&log$=nucltop&blast_rank=1&RID=053B52BE01R) | 552 | 99.44% |
| 68 | First week | WRND Peak | ON790361 | *Cladosporium ramotenellum* | [OM236781.1](https://www.ncbi.nlm.nih.gov/nucleotide/OM236781.1?report=genbank&log$=nucltop&blast_rank=2&RID=5XE76JHR016) | 534 | 99.22% |
| 69 | First week | MSRHD Peak | ON790323 | *Cladosporium cladosporioides* | [OM237197.1](https://www.ncbi.nlm.nih.gov/nucleotide/OM237197.1?report=genbank&log$=nucltop&blast_rank=1&RID=ZZ79H60U013) | 533 | 99.62% |
| 70 | First week | MSRHD Peak | ON790324 | *Cladosporium uredinicola* | [ON790324.1](https://www.ncbi.nlm.nih.gov/nucleotide/ON790324.1?report=genbank&log$=nucltop&blast_rank=1&RID=7SC6FVVK013) | 528 | 100.00% |
| 71 | First week | MSRHD Peak | ON790325 | *Alternaria tamaricis* | [MW009009.1](https://www.ncbi.nlm.nih.gov/nucleotide/MW009009.1?report=genbank&log$=nucltop&blast_rank=2&RID=ZZB1RN3E01R) | 545 | 99.81% |
| 72 | First week | MSRHD Peak | ON790326 | *Periconia byssoides* | [OM237198.1](https://www.ncbi.nlm.nih.gov/nucleotide/OM237198.1?report=genbank&log$=nucltop&blast_rank=2&RID=ZZCKYE9P016) | 550 | 99.26% |
| 73 | First week | MSRHD Peak | ON790327 | *Alternaria tenuissima* | [KY197939.1](https://www.ncbi.nlm.nih.gov/nucleotide/KY197939.1?report=genbank&log$=nucltop&blast_rank=1&RID=ZZDCF53A016) | 555 | 99.45% |
| 74 | First week | MSRHD Peak | ON790362 | *Cladosporium cladosporioides* | [OM236961.1](https://www.ncbi.nlm.nih.gov/nucleotide/OM236961.1?report=genbank&log$=nucltop&blast_rank=1&RID=053WRPEZ013) | 534 | 99.62% |
| 75 | First week | MSRHD Peak | ON790363 | *Alternaria compacta* | [OM237139.1](https://www.ncbi.nlm.nih.gov/nucleotide/OM237139.1?report=genbank&log$=nucltop&blast_rank=1&RID=054AKED201R) | 542 | 99.81% |
| 76 | First week | MSRHD Peak | ON790364 | *Alternaria compacta* | [OM236937.1](https://www.ncbi.nlm.nih.gov/nucleotide/OM236937.1?report=genbank&log$=nucltop&blast_rank=1&RID=054NHN5X013) | 546 | 99.81% |
| 77 | First week | MSRHD Peak | ON790365 | *Cladosporium cladosporioides* | [OM236704.1](https://www.ncbi.nlm.nih.gov/nucleotide/OM236704.1?report=genbank&log$=nucltop&blast_rank=1&RID=054YNNDZ016) | 524 | 99.61% |
| 78 | First week | XRHD Peak | ON790328 | *Alternaria alternata* | [OM237132.1](https://www.ncbi.nlm.nih.gov/nucleotide/OM237132.1?report=genbank&log$=nucltop&blast_rank=1&RID=ZZEUGU4X013) | 554 | 99.81% |
| 79 | First week | XRHD Peak | ON790329 | *Alternaria tenuissima* | [MW009002.1](https://www.ncbi.nlm.nih.gov/nucleotide/MW009002.1?report=genbank&log$=nucltop&blast_rank=1&RID=01G3FUD301R) | 545 | 100.00% |
| 80 | First week | XRHD Peak | ON790330 | *Cladosporium asperulatum* | [MT786367.1](https://www.ncbi.nlm.nih.gov/nucleotide/MT786367.1?report=genbank&log$=nucltop&blast_rank=1&RID=01GBP4GF016) | 527 | 99.22% |
| 81 | First week | XRHD Peak | ON790331 | *Aureobasidium pullulans* | [MK772062.1](https://www.ncbi.nlm.nih.gov/nucleotide/MK772062.1?report=genbank&log$=nucltop&blast_rank=1&RID=01GJJCJR013) | 575 | 99.44% |
| 82 | First week | XRHD Peak | ON790366 | *Aspergillus fumigatus* | [MN704714.1](https://www.ncbi.nlm.nih.gov/nucleotide/MN704714.1?report=genbank&log$=nucltop&blast_rank=1&RID=055F0JF3016) | 575 | 97.87% |
| 83 | First week | XRHD Peak | ON790367 | *Aspergillus fumigatus* | [MT093256.1](https://www.ncbi.nlm.nih.gov/nucleotide/MT093256.1?report=genbank&log$=nucltop&blast_rank=1&RID=0560NHBW01R) | 570 | 99.12% |
| 84 | First week | XRHD Peak | ON790368 | *Aspergillus fumigatus* | [MN588070.1](https://www.ncbi.nlm.nih.gov/nucleotide/MN588070.1?report=genbank&log$=nucltop&blast_rank=1&RID=0567G7MP013) | 572 | 99.47% |
| 85 | First week | XRHD Peak | ON790369 | *Paecilomyces tabacinus* | [OL871292.1](https://www.ncbi.nlm.nih.gov/nucleotide/OL871292.1?report=genbank&log$=nucltop&blast_rank=1&RID=056RU9BJ01R) | 543 | 97.61% |
| 86 | First week | KRHD Peak | ON790332 | *Alternaria alternata* | [KY419541.1](https://www.ncbi.nlm.nih.gov/nucleotide/KY419541.1?report=genbank&log$=nucltop&blast_rank=2&RID=5XG6ZHWM01N) | 546 | 99.45% |
| 87 | First week | KRHD Peak | ON790333 | *Epicoccum nigrum* | [MH645206.1](https://www.ncbi.nlm.nih.gov/nucleotide/MH645206.1?report=genbank&log$=nucltop&blast_rank=1&RID=01H8X2DY013) | 586 | 99.21% |
| 88 | First week | KRHD Peak | ON790334 | *Periconia byssoides* | [OM237198.1](https://www.ncbi.nlm.nih.gov/nucleotide/OM237198.1?report=genbank&log$=nucltop&blast_rank=2&RID=01HKXHTS01R) | 550 | 98.12% |
| 89 | First week | KRHD Peak | ON790335 | *Alternaria brassicae* | [MW009044.1](https://www.ncbi.nlm.nih.gov/nucleotide/MW009044.1?report=genbank&log$=nucltop&blast_rank=1&RID=021UXH88016) | 555 | 99.63% |
| 90 | First week | KRHD Peak | ON790336 | *Cladosporium tenuissimum* | [OM237136.1](https://www.ncbi.nlm.nih.gov/nucleotide/OM237136.1?report=genbank&log$=nucltop&blast_rank=1&RID=0223P3RB016) | 532 | 99.42% |
| 91 | First week | KRHD Peak | ON790337 | *Neocamarosporium betae* | [OM236794.1](https://www.ncbi.nlm.nih.gov/nucleotide/OM236794.1?report=genbank&log$=nucltop&blast_rank=1&RID=022FWJBN016) | 572 | 98.93% |
| 92 | First week | KRHD Peak | ON790338 | *Alternaria alternata* | [OM236753.1](https://www.ncbi.nlm.nih.gov/nucleotide/OM236753.1?report=genbank&log$=nucltop&blast_rank=1&RID=0234CG6M016) | 553 | 99.81% |
| 93 | First week | KRHD Peak | ON790370 | *Cladosporium tenuissimum* | [OM237136.1](https://www.ncbi.nlm.nih.gov/nucleotide/OM237136.1?report=genbank&log$=nucltop&blast_rank=1&RID=056Y0AZX016) | 532 | 100.00% |
| 94 | First week | KRHD Peak | ON790371 | *Alternaria tamaricis* | [OM237300.1](https://www.ncbi.nlm.nih.gov/nucleotide/OM237300.1?report=genbank&log$=nucltop&blast_rank=2&RID=0574S50U016) | 550 | 99.63% |
| 95 | First week | LRHD Peak | ON790339 | *Cladosporium cladosporioides* | [OM237118.1](https://www.ncbi.nlm.nih.gov/nucleotide/OM237118.1?report=genbank&log$=nucltop&blast_rank=1&RID=0259NT5201R) | 531 | 100.00% |
| 96 | First week | LRHD Peak | ON790340 | *Cladosporium cladosporioides* | [OM237122.1](https://www.ncbi.nlm.nih.gov/nucleotide/OM237122.1?report=genbank&log$=nucltop&blast_rank=1&RID=0263VZMY01R) | 533 | 100.00% |
| 97 | First week | LRHD Peak | ON790341 | *Cladosporium tenuissimum* | [OM236674.1](https://www.ncbi.nlm.nih.gov/nucleotide/OM236674.1?report=genbank&log$=nucltop&blast_rank=1&RID=026NF20X016) | 532 | 99.61% |
| 98 | First week | LRHD Peak | ON790342 | *Cladosporium tenuissimum* | [OM237136.1](https://www.ncbi.nlm.nih.gov/nucleotide/OM237136.1?report=genbank&log$=nucltop&blast_rank=1&RID=026YSZ4B016) | 532 | 99.23% |
| 99 | First week | LRHD Peak | ON790343 | *Filobasidium magnum* | [OM236818.1](https://www.ncbi.nlm.nih.gov/nucleotide/OM236818.1?report=genbank&log$=nucltop&blast_rank=1&RID=02BV7XUC016) | 592 | 95.35% |
| 100 | First week | LRHD Peak | ON790344 | *Alternaria alternata* | [OM236773.1](https://www.ncbi.nlm.nih.gov/nucleotide/OM236773.1?report=genbank&log$=nucltop&blast_rank=1&RID=02C52NYV013) | 544 | 98.11% |
| 101 | First week | LRHD Peak | ON790345 | *Cladosporium tenuissimum* | [OM237120.1](https://www.ncbi.nlm.nih.gov/nucleotide/OM237120.1?report=genbank&log$=nucltop&blast_rank=1&RID=02CH8X87013) | 525 | 99.81% |
| 102 | First week | LRHD Peak | ON790346 | *Cladosporium tenuissimum* | [OM237136.1](https://www.ncbi.nlm.nih.gov/nucleotide/OM237136.1?report=genbank&log$=nucltop&blast_rank=1&RID=04M3FZT6013) | 532 | 99.23% |
| 103 | First week | LRHD Peak | ON790347 | *Alternaria tamaricis* | [OM236768.1](https://www.ncbi.nlm.nih.gov/nucleotide/OM236768.1?report=genbank&log$=nucltop&blast_rank=4&RID=1FC1TDD0013) | 552 | 99.27% |
| 104 | First week | LRHD Peak | ON790348 | *Alternaria alternata* | [OM236755.1](https://www.ncbi.nlm.nih.gov/nucleotide/OM236755.1?report=genbank&log$=nucltop&blast_rank=1&RID=04N0G1XD016) | 558 | 99.63% |
| 105 | First week | LRHD Peak | ON790349 | *Alternaria compacta* | [OM237139.1](https://www.ncbi.nlm.nih.gov/nucleotide/OM237139.1?report=genbank&log$=nucltop&blast_rank=1&RID=04NA7ESG016) | 542 | 99.45% |
| 106 | First week | LRHD Peak | ON790350 | *Alternaria compacta* | [MW008920.1](https://www.ncbi.nlm.nih.gov/nucleotide/MW008920.1?report=genbank&log$=nucltop&blast_rank=1&RID=04NS51DB01R) | 540 | 100.00% |
| 107 | Second week | WRND Off-peak | ON790372 | *Filobasidium magnum* | [OM236818.1](https://www.ncbi.nlm.nih.gov/nucleotide/OM236818.1?report=genbank&log$=nucltop&blast_rank=1&RID=06VPWE45013) | 592 | 96.27% |
| 108 | Second week | WRND Off-peak | ON790373 | *Alternaria tenuissima* | [MG731240.1](https://www.ncbi.nlm.nih.gov/nucleotide/MG731240.1?report=genbank&log$=nucltop&blast_rank=2&RID=06WFH0GK016) | 545 | 99.81% |
| 109 | Second week | WRND Off-peak | ON790405 | *Spencermartinsia viticola* | [KF466504.1](https://www.ncbi.nlm.nih.gov/nucleotide/KF466504.1?report=genbank&log$=nucltop&blast_rank=2&RID=21DENRME016) | 559 | 99.04% |
| 110 | Second week | MSRHD Off-peak | ON790374 | *Dothideomycetes* sp. | [GQ153260.1](https://www.ncbi.nlm.nih.gov/nucleotide/GQ153260.1?report=genbank&log$=nucltop&blast_rank=2&RID=5XJXWSEV013) | 1055 | 99.02% |
| 111 | Second week | MSRHD Off-peak | ON790375 | *Aureobasidium iranianum* | [OM236763.1](https://www.ncbi.nlm.nih.gov/nucleotide/OM236763.1?report=genbank&log$=nucltop&blast_rank=1&RID=06XX26DE013) | 574 | 99.45% |
| 112 | Second week | MSRHD Off-peak | ON790376 | *Curvularia kusanoi* | [MF061766.1](https://www.ncbi.nlm.nih.gov/nucleotide/MF061766.1?report=genbank&log$=nucltop&blast_rank=1&RID=06Y60HWH013) | 507 | 99.60% |
| 113 | Second week | MSRHD Off-peak | ON790377 | *Spencermartinsia viticola* | [JX241669.1](https://www.ncbi.nlm.nih.gov/nucleotide/JX241669.1?report=genbank&log$=nucltop&blast_rank=1&RID=070XNMMF01R) | 532 | 99.62% |
| 114 | Second week | MSRHD Off-peak | ON790378 | *Ascochyta medicaginicola* | [OM237193.1](https://www.ncbi.nlm.nih.gov/nucleotide/OM237193.1?report=genbank&log$=nucltop&blast_rank=1&RID=071EKTAX016) | 527 | 100.00% |
| 115 | Second week | MSRHD Off-peak | ON790379 | *Dothiorella viticola* | [KY385661.1](https://www.ncbi.nlm.nih.gov/nucleotide/KY385661.1?report=genbank&log$=nucltop&blast_rank=1&RID=07202319016) | 554 | 99.42% |
| 116 | Second week | MSRHD Off-peak | ON790380 | *Paraconiothyrium hawaiiense* | [HM751092.1](https://www.ncbi.nlm.nih.gov/nucleotide/HM751092.1?report=genbank&log$=nucltop&blast_rank=2&RID=5XKBUZEE016) | 1059 | 96.55% |
| 117 | Second week | MSRHD Off-peak | ON790381 | *Paraconiothyrium hawaiiense* | [HM751092.1](https://www.ncbi.nlm.nih.gov/nucleotide/HM751092.1?report=genbank&log$=nucltop&blast_rank=2&RID=5XM6X88J013) | 1059 | 94.15% |
| 118 | Second week | MSRHD Off-peak | ON790382 | *Paraconiothyrium hawaiiense* | [HM751092.1](https://www.ncbi.nlm.nih.gov/nucleotide/HM751092.1?report=genbank&log$=nucltop&blast_rank=2&RID=5XMR9G22013) | 1059 | 95.76% |
| 119  120 | Second week  Second week | XRHD Off-peak  KRHD Off-peak | ON790383  ON790384 | *Aspergillus caesiellus*  *Penicillium oxalicum* | [KP269016.1](https://www.ncbi.nlm.nih.gov/nucleotide/KP269016.1?report=genbank&log$=nucltop&blast_rank=1&RID=098R8FNA013)  [JN226949.1](https://www.ncbi.nlm.nih.gov/nucleotide/JN226949.1?report=genbank&log$=nucltop&blast_rank=2&RID=098ZPUHU013) | 589  548 | 99.45%  99.82% |
| 121 | Second week | KRHD Off-peak | ON790385 | *Alternaria alternata* | [MT093259.1](https://www.ncbi.nlm.nih.gov/nucleotide/MT093259.1?report=genbank&log$=nucltop&blast_rank=1&RID=099PAPYS01R) | 546 | 99.44% |
| 122 | Second week | KRHD Off-peak | ON790386 | *Alternaria alternata* | [MT093259.1](https://www.ncbi.nlm.nih.gov/nucleotide/MT093259.1?report=genbank&log$=nucltop&blast_rank=1&RID=09AH1PPA01R) | 546 | 99.63% |
| 123 | Second week | KRHD Off-peak | ON790406 | *Alternaria compacta* | [OM236771.1](https://www.ncbi.nlm.nih.gov/nucleotide/OM236771.1?report=genbank&log$=nucltop&blast_rank=1&RID=21K78YPB013) | 549 | 99.63% |
| 124 | Second week | KRHD Off-peak | ON790407 | *Penicillium oxalicum* | [MK281571.1](https://www.ncbi.nlm.nih.gov/nucleotide/MK281571.1?report=genbank&log$=nucltop&blast_rank=1&RID=21KP0K51013) | 565 | 99.46% |
| 125 | Second week | KRHD Off-peak | ON790408 | *Penicillium oxalicum* | [KC880081.1](https://www.ncbi.nlm.nih.gov/nucleotide/KC880081.1?report=genbank&log$=nucltop&blast_rank=2&RID=21M7726S013) | 564 | 99.63% |
| 126 | Second week | LRHD Off-peak | ON790387 | *Cladosporium sphaerospermum* | [KP269048.1](https://www.ncbi.nlm.nih.gov/nucleotide/KP269048.1?report=genbank&log$=nucltop&blast_rank=1&RID=09AS6YUB013) | 511 | 92.08% |
| 127 | Second week | LRHD Off-peak | ON790388 | *Aspergillus fumigatus* | [OM372895.1](https://www.ncbi.nlm.nih.gov/nucleotide/OM372895.1?report=genbank&log$=nucltop&blast_rank=2&RID=19YDMJFY013) | 570 | 99.82% |
| 128 | Second week | WRND Peak | ON790389 | *Cladosporium* sp.  *Cladosporium cladosporioides* | [KX761856.1](https://www.ncbi.nlm.nih.gov/nucleotide/KX761856.1?report=genbank&log$=nucltop&blast_rank=1&RID=19YTXTP8016)  [OM236686.1](https://www.ncbi.nlm.nih.gov/nucleotide/OM236686.1?report=genbank&log$=nucltop&blast_rank=3&RID=19YTXTP8016) | 536  534 | 99.24%  99.61% |
| 129 | Second week | WRND Peak | ON790390 | *Cladosporium cladosporioides* | [AB369498.1](https://www.ncbi.nlm.nih.gov/nucleotide/AB369498.1?report=genbank&log$=nucltop&blast_rank=1&RID=19ZFRZ2801R) | 505 | 90.20% |
| 130 | Second week | WRND Peak | ON790391 | *Cladosporium cladosporioides* | [OM236703.1](https://www.ncbi.nlm.nih.gov/nucleotide/OM236703.1?report=genbank&log$=nucltop&blast_rank=3&RID=21139GAS016) | 531 | 99.61% |
| 131 | Second week | WRND Peak | ON790392 | *Cladosporium cladosporioides* | [MF061760.1](https://www.ncbi.nlm.nih.gov/nucleotide/MF061760.1?report=genbank&log$=nucltop&blast_rank=3&RID=211F26V5013) | 521 | 100.00% |
| 132 | Second week | WRND Peak | ON790409 | *Cladosporium oxysporum* | [JQ775499.1](https://www.ncbi.nlm.nih.gov/nucleotide/JQ775499.1?report=genbank&log$=nucltop&blast_rank=2&RID=21NPZ7P3016) | 525 | 96.91% |
| 133 | Second week | WRND Peak | ON790410 | *Aspergillus oryzae* | [MT306000.1](https://www.ncbi.nlm.nih.gov/nucleotide/MT306000.1?report=genbank&log$=nucltop&blast_rank=2&RID=21NYV59D013) | 567 | 98.36% |
| 134 | Second week | WRND Peak | ON790411 | *Cladosporium oxysporum* | [OM236950.1](https://www.ncbi.nlm.nih.gov/nucleotide/OM236950.1?report=genbank&log$=nucltop&blast_rank=1&RID=21P878F8016) | 535 | 99.61% |
| 135 | Second week | WRND Peak | ON790412 | *Cladosporium cladosporioides* | [OM236686.1](https://www.ncbi.nlm.nih.gov/nucleotide/OM236686.1?report=genbank&log$=nucltop&blast_rank=1&RID=21PPJ0YE01R) | 534 | 100.00% |
| 136 | Second week | WRND Peak | ON790413 | *Cladosporium cladosporioides* | [OM236984.1](https://www.ncbi.nlm.nih.gov/nucleotide/OM236984.1?report=genbank&log$=nucltop&blast_rank=2&RID=21R26TWA013) | 531 | 99.81% |
| 137 | Second week | WRND Peak | ON790414 | *Cladosporium oxysporum* | [OM236688.1](https://www.ncbi.nlm.nih.gov/nucleotide/OM236688.1?report=genbank&log$=nucltop&blast_rank=1&RID=21RACPX0016) | 521 | 99.80% |
| 138 | Second week | MSRHD Peak | ON790393 | *Alternaria tenuissima* | [JX406499.1](https://www.ncbi.nlm.nih.gov/nucleotide/JX406499.1?report=genbank&log$=nucltop&blast_rank=3&RID=21276WZT016) | 545 | 99.44% |
| 139 | Second week | MSRHD Peak | ON790394 | *Cladosporium cladosporioides* | [OM237204.1](https://www.ncbi.nlm.nih.gov/nucleotide/OM237204.1?report=genbank&log$=nucltop&blast_rank=1&RID=212MC0UX01R) | 525 | 99.22% |
| 140 | Second week | MSRHD Peak | ON790395 | *Cladosporium cladosporioides* | [MW113635.1](https://www.ncbi.nlm.nih.gov/nucleotide/MW113635.1?report=genbank&log$=nucltop&blast_rank=3&RID=2133TJ1C01R) | 669 | 100.00% |
| 141 | Second week | MSRHD Peak | ON790396 | *Dothideomycetes* sp.  *Phoma betae* | [MK100231.1](https://www.ncbi.nlm.nih.gov/nucleotide/MK100231.1?report=genbank&log$=nucltop&blast_rank=3&RID=213WBDH5013)  [MK460862.1](https://www.ncbi.nlm.nih.gov/nucleotide/MK460862.1?report=genbank&log$=nucltop&blast_rank=1&RID=213WBDH5013) | 574  624 | 99.44%  99.08% |
| 142 | Second week | MSRHD Peak | ON790397 | *Nothophoma quercina* | [KX064984.1](https://www.ncbi.nlm.nih.gov/nucleotide/KX064984.1?report=genbank&log$=nucltop&blast_rank=2&RID=5XN3BRYF013) | 518 | 100.00% |
| 143 | Second week | MSRHD Peak | ON790398 | *Alternaria compacta* | [MW008929.1](https://www.ncbi.nlm.nih.gov/nucleotide/MW008929.1?report=genbank&log$=nucltop&blast_rank=1&RID=216MK3WJ016) | 544 | 99.63% |
| 144 | Second week | MSRHD Peak | ON790399 | *Aspergillus versicolor* | [MT497452.1](https://www.ncbi.nlm.nih.gov/nucleotide/MT497452.1?report=genbank&log$=nucltop&blast_rank=1&RID=2173SAV6013) | 856 | 99.45% |
| 145 | Second week | MSRHD Peak | ON790400 | *Phoma medicaginis* | [KT192415.1](https://www.ncbi.nlm.nih.gov/nucleotide/KT192415.1?report=genbank&log$=nucltop&blast_rank=1&RID=217PRS9U013) | 543 | 99.41% |
| 146 | Second week | MSRHD Peak | ON790415 | *Cladosporium halotolerans* | [OM236856.1](https://www.ncbi.nlm.nih.gov/nucleotide/OM236856.1?report=genbank&log$=nucltop&blast_rank=1&RID=21RT78D9016) | 508 | 98.00% |
| 147 | Second week | MSRHD Peak | ON790416 | *Aspergillus flavus* | [MT529930.1](https://www.ncbi.nlm.nih.gov/nucleotide/MT529930.1?report=genbank&log$=nucltop&blast_rank=1&RID=21S0MBJU01R) | 556 | 97.62% |
| 148 | Second week | XRHD Peak | ON790401 | *Alternaria alternata* | [OM236694.1](https://www.ncbi.nlm.nih.gov/nucleotide/OM236694.1?report=genbank&log$=nucltop&blast_rank=1&RID=2192MU4F016) | 545 | 99.81% |
| 149 | Second week | KRHD Peak | ON790402 | *Alternaria* sp.  *Alternaria alternata* | [OM236935.1](https://www.ncbi.nlm.nih.gov/nucleotide/OM236935.1?report=genbank&log$=nucltop&blast_rank=1&RID=21ACJ9CT016)  [MG711600.1](https://www.ncbi.nlm.nih.gov/nucleotide/MG711600.1?report=genbank&log$=nucltop&blast_rank=3&RID=21ACJ9CT016) | 547  542 | 99.63%  99.45% |
| 150 | Second week | KRHD Peak | ON790403 | *Moesziomyces bullatus* | [MK024203.1](https://www.ncbi.nlm.nih.gov/nucleotide/MK024203.1?report=genbank&log$=nucltop&blast_rank=1&RID=21B1S3U0013) | 755 | 100.00% |
| 151 | Second week | KRHD Peak | ON790417 | *Aspergillus* sp.  *Aspergillus versicolor* | [KX928747.1](https://www.ncbi.nlm.nih.gov/nucleotide/KX928747.1?report=genbank&log$=nucltop&blast_rank=3&RID=5XNEA96E013)  [KP994296.1](https://www.ncbi.nlm.nih.gov/nucleotide/KP994296.1?report=genbank&log$=nucltop&blast_rank=4&RID=5XNEA96E013) | 613  576 | 99.45%  99.45% |
| 152 | Second week | KRHD Peak | ON790418 | *Coprinopsis* sp.  *Coprinopsis atramentaria* | [MF579594.1](https://www.ncbi.nlm.nih.gov/nucleotide/MF579594.1?report=genbank&log$=nucltop&blast_rank=1&RID=21T277NJ016)  [MG132086.1](https://www.ncbi.nlm.nih.gov/nucleotide/MG132086.1?report=genbank&log$=nucltop&blast_rank=2&RID=21T277NJ016) | 671  681 | 99.54%  99.38% |
| 153 | Second week | KRHD Peak | ON790419 | *Aspergillus versicolor* | [MK732128.1](https://www.ncbi.nlm.nih.gov/nucleotide/MK732128.1?report=genbank&log$=nucltop&blast_rank=1&RID=21T91FRG016) | 559 | 99.26% |
| 154 | Second week | KRHD Peak | ON790420 | *Aspergillus versicolor* | [MK732128.1](https://www.ncbi.nlm.nih.gov/nucleotide/MK732128.1?report=genbank&log$=nucltop&blast_rank=1&RID=21TN4B6P016) | 559 | 98.89% |
| 155 | Second week | KRHD Peak | ON790421 | *Aspergillus* sp.  *Aspergillus creber* | [MK605980.1](https://www.ncbi.nlm.nih.gov/nucleotide/MK605980.1?report=genbank&log$=nucltop&blast_rank=1&RID=21U282UE016)  [LN898693.1](https://www.ncbi.nlm.nih.gov/nucleotide/LN898693.1?report=genbank&log$=nucltop&blast_rank=2&RID=21U282UE016) | 760  760 | 99.44%  99.44% |
| 156 | Second week | KRHD Peak | ON790422 | *Aspergillus sydowii* | [MT530154.1](https://www.ncbi.nlm.nih.gov/nucleotide/MT530154.1?report=genbank&log$=nucltop&blast_rank=1&RID=21VXBPB5013) | 580 | 99.81% |
| 157 | Second week | KRHD Peak | ON790423 | *Aspergillus* sp.  *Aspergillus creber* | [MK605980.1](https://www.ncbi.nlm.nih.gov/nucleotide/MK605980.1?report=genbank&log$=nucltop&blast_rank=1&RID=21W8EEEV01R)  [LN898693.1](https://www.ncbi.nlm.nih.gov/nucleotide/LN898693.1?report=genbank&log$=nucltop&blast_rank=2&RID=21W8EEEV01R) | 760  760 | 99.44%  99.44% |
| 158 | Second week | KRHD Peak | ON790424 | *Alternaria brassicicola* | [MW851892.1](https://www.ncbi.nlm.nih.gov/nucleotide/MW851892.1?report=genbank&log$=nucltop&blast_rank=1&RID=21WGXEAU01R) | 547 | 99.26% |
| 159 | Second week | KRHD Peak | ON790425 | *Aspergillus creber* | [OL616067.1](https://www.ncbi.nlm.nih.gov/nucleotide/OL616067.1?report=genbank&log$=nucltop&blast_rank=3&RID=5V200N0G016) | 564 | 99.44% |
| 160 | Second week | KRHD Peak | ON790426 | *Aspergillus creber* | [LN898693.1](https://www.ncbi.nlm.nih.gov/nucleotide/LN898693.1?report=genbank&log$=nucltop&blast_rank=2&RID=21X6WP4T013) | 760 | 98.90% |
| 161 | Second week | KRHD Peak | ON790427 | *Aspergillus creber* | [LN898693.1](https://www.ncbi.nlm.nih.gov/nucleotide/LN898693.1?report=genbank&log$=nucltop&blast_rank=2&RID=21XDRCP8013) | 760 | 97.15% |
| 162 | Second week | KRHD Peak | ON790428 | *Aspergillus versicolor* | [AM883156.1](https://www.ncbi.nlm.nih.gov/nucleotide/AM883156.1?report=genbank&log$=nucltop&blast_rank=4&RID=21Y8PG72013) | 1151 | 99.26% |
| 163 | Second week | KRHD Peak | ON790429 | *Aspergillus versicolor* | [KX302039.1](https://www.ncbi.nlm.nih.gov/nucleotide/KX302039.1?report=genbank&log$=nucltop&blast_rank=1&RID=21Y4CATW016) | 552 | 99.44% |
| 164 | Second week | KRHD Peak | ON790430 | *Aspergillus versicolor* | [KX302039.1](https://www.ncbi.nlm.nih.gov/nucleotide/KX302039.1?report=genbank&log$=nucltop&blast_rank=1&RID=21XZSN3M01R) | 552 | 99.26% |
| 165 | Second week | KRHD Peak | ON790431 | *Aspergillus versicolor* | [KX302040.1](https://www.ncbi.nlm.nih.gov/nucleotide/KX302040.1?report=genbank&log$=nucltop&blast_rank=1&RID=21XUFPZ901R) | 542 | 99.62% |
| 166 | Second week | LRHD Peak | ON790404 | *Alternaria alternata* | [OM236694.1](https://www.ncbi.nlm.nih.gov/nucleotide/OM236694.1?report=genbank&log$=nucltop&blast_rank=1&RID=21D20V6Y01R) | 545 | 99.63% |
| 167 | Second week | LRHD Peak | ON790432 | *Aspergillus flavus* | [MZ723884.1](https://www.ncbi.nlm.nih.gov/nucleotide/MZ723884.1?report=genbank&log$=nucltop&blast_rank=2&RID=21XNCTVP016) | 578 | 98.94% |
| 168 | Third week | WRND Off-peak | ON790433 | *Coprinellus radians* | [KP900252.1](https://www.ncbi.nlm.nih.gov/nucleotide/KP900252.1?report=genbank&log$=nucltop&blast_rank=2&RID=2SH0K0KK013) | 658 | 99.84% |
| 169 | Third week | WRND Off-peak | ON790434 | *Penicillium oxalicum* | [MW689664.1](https://www.ncbi.nlm.nih.gov/nucleotide/MW689664.1?report=genbank&log$=nucltop&blast_rank=2&RID=2SHDNA1K016) | 556 | 100.00% |
| 170 | Third week | MSRHD Off-peak | ON790435 | *Coprinellus radians* | [MN547379.1](https://www.ncbi.nlm.nih.gov/nucleotide/MN547379.1?report=genbank&log$=nucltop&blast_rank=1&RID=2SHV9XNR016) | 697 | 100.00% |
| 171 | Third week | MSRHD Off-peak | ON790436 | *Coprinellus radians* | [MN547379.1](https://www.ncbi.nlm.nih.gov/nucleotide/MN547379.1?report=genbank&log$=nucltop&blast_rank=1&RID=2SK2CSJW016) | 697 | 99.68% |
| 172 | Third week | MSRHD Off-peak | ON790437 | *Schizophyllum commune* | [MF061788.1](https://www.ncbi.nlm.nih.gov/nucleotide/MF061788.1?report=genbank&log$=nucltop&blast_rank=2&RID=2SKPDHFJ016) | 606 | 99.83% |
| 173 | Third week | MSRHD Off-peak | ON790438 | *Ceratobasidium* sp. | [MH483971.1](https://www.ncbi.nlm.nih.gov/nucleotide/MH483971.1?report=genbank&log$=nucltop&blast_rank=2&RID=2SM21SED01R) | 639 | 99.83% |
| 174 | Third week | MSRHD Off-peak | ON790439 | *Coprinellus radians* | [MN398974.1](https://www.ncbi.nlm.nih.gov/nucleotide/MN398974.1?report=genbank&log$=nucltop&blast_rank=1&RID=2SP9KPVB01R) | 666 | 99.84% |
| 175 | Third week | MSRHD Off-peak | ON790440 | *Efibula matsuensis* | [NR175181.1](https://www.ncbi.nlm.nih.gov/nucleotide/NR_175181.1?report=genbank&log$=nucltop&blast_rank=2&RID=333ZZ931016) | 627 | 73.66% |
| 176 | Third week | MSRHD Off-peak | ON790441 | *Aureobasidium pullulans* | [ON790441.1](https://www.ncbi.nlm.nih.gov/nucleotide/KP027644.1?report=genbank&log$=nucltop&blast_rank=1&RID=334KHURB016) | 534 | 100.00% |
| 177 | Third week | MSRHD Off-peak | ON790442 | *Coprinellus radians* | [KP900252.1](https://www.ncbi.nlm.nih.gov/nucleotide/KP900252.1?report=genbank&log$=nucltop&blast_rank=1&RID=7RP2YEMC01N) | 658 | 96.49% |
| 178 | Third week | MSRHD Off-peak | ON790443 | *Coprinellus radians* | [MN398974.1](https://www.ncbi.nlm.nih.gov/nucleotide/MN398974.1?report=genbank&log$=nucltop&blast_rank=2&RID=33548W27013) | 666 | 99.84% |
| 179 | Third week | MSRHD Off-peak | ON790444 | *Russulales* sp. | [MK304378.1](https://www.ncbi.nlm.nih.gov/nucleotide/MK304378.1?report=genbank&log$=nucltop&blast_rank=7&RID=336STPEP01R) | 568 | 98.47% |
| 180 | Third week | MSRHD Off-peak | ON790445 | *Stagonospora* sp. | [OM236799.1](https://www.ncbi.nlm.nih.gov/nucleotide/OM236799.1?report=genbank&log$=nucltop&blast_rank=1&RID=3376FUA7016) | 579 | 99.08% |
| 181 | Third week | XRHD Off-peak | ON790446 | *Gloeophyllum trabeum* | [KJ995949.1](https://www.ncbi.nlm.nih.gov/nucleotide/KJ995949.1?report=genbank&log$=nucltop&blast_rank=3&RID=337SRW1J016) | 799 | 99.82% |
| 182 | Third week | XRHD Off-peak | ON790447 | *Coprinellus radians* | [MN547379.1](https://www.ncbi.nlm.nih.gov/nucleotide/MN547379.1?report=genbank&log$=nucltop&blast_rank=6&RID=3387JKEM013) | 697 | 100.00% |
| 183 | Third week | XRHD Off-peak | ON790448 | *Periconia* sp. | [LC586223.1](https://www.ncbi.nlm.nih.gov/nucleotide/LC586223.1?report=genbank&log$=nucltop&blast_rank=3&RID=35M09DKZ016) | 598 | 99.61% |
| 184 | Third week | XRHD Off-peak | ON790449 | *Periconia pseudobyssoides* | [MT859918.1](https://www.ncbi.nlm.nih.gov/nucleotide/MT859918.1?report=genbank&log$=nucltop&blast_rank=1&RID=35MXYNB9016) | 551 | 99.81% |
| 185 | Third week | XRHD Off-peak | ON790450 | *Setosphaeria turcica* | [KJ922735.1](https://www.ncbi.nlm.nih.gov/nucleotide/KJ922735.1?report=genbank&log$=nucltop&blast_rank=1&RID=35NGZSZ8016) | 540 | 100.00% |
| 186 | Third week | XRHD Off-peak | ON790451 | *Coriolopsis trogii* | [MW554374.1](https://www.ncbi.nlm.nih.gov/nucleotide/MW554374.1?report=genbank&log$=nucltop&blast_rank=1&RID=3850T8PY016) | 654 | 99.18% |
| 187 | Third week | XRHD Off-peak | ON790452 | *Aureobasidium pullulans* | [MK772062.1](https://www.ncbi.nlm.nih.gov/nucleotide/MK772062.1?report=genbank&log$=nucltop&blast_rank=1&RID=3857DD9P016) | 575 | 98.91% |
| 188 | Third week | XRHD Off-peak | ON790453 | *Aureobasidium pullulans* | [MT786361.1](https://www.ncbi.nlm.nih.gov/nucleotide/MT786361.1?report=genbank&log$=nucltop&blast_rank=1&RID=386JY7DX013) | 559 | 99.63% |
| 189 | Third week | XRHD Off-peak | ON790454 | *Cercospora capsici* | [KM357318.1](https://www.ncbi.nlm.nih.gov/nucleotide/KM357318.1?report=genbank&log$=nucltop&blast_rank=1&RID=385SF49M016) | 502 | 99.80% |
| 190 | Third week | XRHD Off-peak | ON790455 | *Aspergillus fumigatus* | [MK713446.1](https://www.ncbi.nlm.nih.gov/nucleotide/MK713446.1?report=genbank&log$=nucltop&blast_rank=2&RID=386YTTWJ016) | 548 | 93.00% |
| 191 | Third week | XRHD Off-peak | ON790456 | *Aspergillus fumigatus* | [MW689676.1](https://www.ncbi.nlm.nih.gov/nucleotide/MW689676.1?report=genbank&log$=nucltop&blast_rank=1&RID=387GAJ9J016) | 566 | 99.47% |
| 192 | Third week | KRHD Off-peak | ON790457 | *Cladosporium tenuissimum* | [MK370679.1](https://www.ncbi.nlm.nih.gov/nucleotide/MK370679.1?report=genbank&log$=nucltop&blast_rank=1&RID=387ZRGC5016) | 526 | 98.85% |
| 193 | Third week | KRHD Off-peak | ON790458 | *Paraconiothyrium* sp.  *Paraconiothyrium brasiliense* | [KX247121.1](https://www.ncbi.nlm.nih.gov/nucleotide/KX247121.1?report=genbank&log$=nucltop&blast_rank=3&RID=3894FR3S016)  [MH790214.1](https://www.ncbi.nlm.nih.gov/nucleotide/MH790214.1?report=genbank&log$=nucltop&blast_rank=4&RID=3894FR3S016) | 537  582 | 99.63%  98.04% |
| 194 | Third week | KRHD Off-peak | ON790459 | *Setosphaeria turcica* | [LT631336.1](https://www.ncbi.nlm.nih.gov/nucleotide/LT631336.1?report=genbank&log$=nucltop&blast_rank=1&RID=389V4HAU013) | 718 | 82.79% |
| 195 | Third week | KRHD Off-peak | ON790460 | *Schizophyllum commune* | [ON153200.1](https://www.ncbi.nlm.nih.gov/nucleotide/ON153200.1?report=genbank&log$=nucltop&blast_rank=2&RID=7JET339001N) | 599 | 99.66% |
| 196 | Third week | LRHD Off-peak | ON790461 | *Cladosporium tenuissimum* | [OM236705.1](https://www.ncbi.nlm.nih.gov/nucleotide/OM236705.1?report=genbank&log$=nucltop&blast_rank=1&RID=3901MKSZ016) | 528 | 99.61% |
| 197 | Third week | LRHD Off-peak | ON790462 | *Efibula matsuensis* | [MZ636957.1](https://www.ncbi.nlm.nih.gov/nucleotide/MZ636957.1?report=genbank&log$=nucltop&blast_rank=1&RID=390YXGFW013) | 624 | 99.83% |
| 198 | Third week | LRHD Off-peak | ON790463 | *Cladosporium cladosporioides* | [MF061760.1](https://www.ncbi.nlm.nih.gov/nucleotide/MF061760.1?report=genbank&log$=nucltop&blast_rank=2&RID=391HHB1J016) | 521 | 99.61% |
| 199 | Third week | LRHD Off-peak | ON790464 | *Coprinellus radians* | [MN547379.1](https://www.ncbi.nlm.nih.gov/nucleotide/MN547379.1?report=genbank&log$=nucltop&blast_rank=1&RID=391YHFKG013) | 697 | 100.00% |
| 200 | Third week | WRND Peak | ON790465 | *Cladosporium cladosporioides* | [MF135505.1](https://www.ncbi.nlm.nih.gov/nucleotide/MF135505.1?report=genbank&log$=nucltop&blast_rank=1&RID=3929HUA9016) | 526 | 99.61% |
| 201 | Third week | WRND Peak | ON790466 | *Alternaria tenuissima* | [MW008902.1](https://www.ncbi.nlm.nih.gov/nucleotide/MW008902.1?report=genbank&log$=nucltop&blast_rank=1&RID=392TJ113013) | 540 | 99.63% |
| 202 | Third week | WRND Peak | ON790467 | *Alternaria compacta* | [MW009019.1](https://www.ncbi.nlm.nih.gov/nucleotide/MW009019.1?report=genbank&log$=nucltop&blast_rank=1&RID=39361KEF013) | 553 | 99.63% |
| 203 | Third week | WRND Peak | ON790468 | *Cladosporium cladosporioides* | [KX815294.1](https://www.ncbi.nlm.nih.gov/nucleotide/KX815294.1?report=genbank&log$=nucltop&blast_rank=1&RID=393YYZYH016) | 597 | 99.23% |
| 204 | Third week | MSRHD Peak | ON790469 | *Alternaria alternata* | [ON790469.1](https://www.ncbi.nlm.nih.gov/nucleotide/ON790469.1?report=genbank&log$=nucltop&blast_rank=1&RID=6VETTXJU013) | 548 | 100.00% |
| 205 | Third week | MSRHD Peak | ON790470 | *Cladosporium cladosporioides* | [OM236986.1](https://www.ncbi.nlm.nih.gov/nucleotide/OM236986.1?report=genbank&log$=nucltop&blast_rank=1&RID=394UHTDA016) | 522 | 99.81% |
| 206 | Third week | MSRHD Peak | ON790471 | *Alternaria compacta* | [MW008904.1](https://www.ncbi.nlm.nih.gov/nucleotide/MW008904.1?report=genbank&log$=nucltop&blast_rank=1&RID=3952A6CG013) | 542 | 100.00% |
| 207 | Third week | MSRHD Peak | ON790472 | *Alternaria alternata* | [KU533841.1](https://www.ncbi.nlm.nih.gov/nucleotide/KU533841.1?report=genbank&log$=nucltop&blast_rank=3&RID=3958XRXW016) | 547 | 99.44% |
| 208 | Third week | MSRHD Peak | ON790473 | *Alternaria alternata* | [KX588109.1](https://www.ncbi.nlm.nih.gov/nucleotide/KX588109.1?report=genbank&log$=nucltop&blast_rank=2&RID=395S934W013) | 547 | 99.81% |
| 209 | Third week | MSRHD Peak | ON790474 | *Alternaria alternata* | [MZ930190.1](https://www.ncbi.nlm.nih.gov/nucleotide/MZ930190.1?report=genbank&log$=nucltop&blast_rank=2&RID=396EKZP7016) | 554 | 100.00% |
| 210 | Third week | MSRHD Peak | ON790475 | *Alternaria brassicae* | [MF356574.1](https://www.ncbi.nlm.nih.gov/nucleotide/MF356574.1?report=genbank&log$=nucltop&blast_rank=2&RID=396XS6R7013) | 568 | 99.26% |
| 211 | Third week | MSRHD Peak | ON790476 | *Fusarium solani* | [MH612969.1](https://www.ncbi.nlm.nih.gov/nucleotide/MH612969.1?report=genbank&log$=nucltop&blast_rank=1&RID=3JJYKSBK013) | 582 | 99.63% |
| 212 | Third week | XRHD Peak | ON790477 | *Alternaria alternata* | [MG755752.1](https://www.ncbi.nlm.nih.gov/nucleotide/MG755752.1?report=genbank&log$=nucltop&blast_rank=1&RID=3JKEDGC2013) | 567 | 99.81% |
| 213 | Third week | XRHD Peak | ON790478 | *Alternaria compacta* | [MW008933.1](https://www.ncbi.nlm.nih.gov/nucleotide/MW008933.1?report=genbank&log$=nucltop&blast_rank=1&RID=40WTGKNB013) | 540 | 100.00% |
| 214 | Third week | XRHD Peak | ON790479 | *Alternaria alternata* | [OM236693.1](https://www.ncbi.nlm.nih.gov/nucleotide/OM236693.1?report=genbank&log$=nucltop&blast_rank=2&RID=40XC6NH2013) | 544 | 99.44% |
| 215 | Third week | XRHD Peak | ON790480 | *Penicillium oxalicum* | [MZ227287.1](https://www.ncbi.nlm.nih.gov/nucleotide/MZ227287.1?report=genbank&log$=nucltop&blast_rank=1&RID=40XT4PTY016) | 564 | 99.64% |
| 216 | Third week | XRHD Peak | ON790481 | *Penicillium oxalicum* | [MN103553.1](https://www.ncbi.nlm.nih.gov/nucleotide/MN103553.1?report=genbank&log$=nucltop&blast_rank=1&RID=40Y8BHYJ016) | 567 | 99.45% |
| 217 | Third week | XRHD Peak | ON790482 | *Penicillium oxalicum* | [KY400080.1](https://www.ncbi.nlm.nih.gov/nucleotide/KY400080.1?report=genbank&log$=nucltop&blast_rank=1&RID=40YY6A31016) | 574 | 99.82% |
| 218 | Third week | XRHD Peak | ON790483 | *Alternaria tenuissima* | [MW008898.1](https://www.ncbi.nlm.nih.gov/nucleotide/MW008898.1?report=genbank&log$=nucltop&blast_rank=2&RID=40ZX9Z8W01R) | 557 | 99.81% |
| 219 | Third week | XRHD Peak | ON790484 | *Alternaria compacta* | [KX074003.1](https://www.ncbi.nlm.nih.gov/nucleotide/KX074003.1?report=genbank&log$=nucltop&blast_rank=1&RID=4109GFMW016) | 564 | 99.45% |
| 220 | Third week | XRHD Peak | ON790485 | *Nothophoma spiraeae* | [OM287410.1](https://www.ncbi.nlm.nih.gov/nucleotide/OM287410.1?report=genbank&log$=nucltop&blast_rank=3&RID=410TURXY016) | 546 | 99.21% |
| 221 | Third week | XRHD Peak | ON790486 | *Nothophoma spiraeae* | [OM237257.1](https://www.ncbi.nlm.nih.gov/nucleotide/OM237257.1?report=genbank&log$=nucltop&blast_rank=1&RID=5DYVE3KN013) | 531 | 99.61% |
| 222 | Third week | XRHD Peak | ON790487 | *Penicillium oxalicum* | [KY400080.1](https://www.ncbi.nlm.nih.gov/nucleotide/KY400080.1?report=genbank&log$=nucltop&blast_rank=1&RID=5DZ5DC3U013) | 574 | 99.64% |
| 223 | Third week | XRHD Peak | ON790488 | *Alternaria compacta* | [OM236771.1](https://www.ncbi.nlm.nih.gov/nucleotide/OM236771.1?report=genbank&log$=nucltop&blast_rank=1&RID=5DZEESEG016) | 549 | 99.63% |
| 224 | Third week | XRHD Peak | ON790489 | *Talaromyces verruculosus* | [OM237165.1](https://www.ncbi.nlm.nih.gov/nucleotide/OM237165.1?report=genbank&log$=nucltop&blast_rank=1&RID=5E06Y0GR016) | 558 | 98.33% |
| 225 | Third week | XRHD Peak | ON790490 | *Alternaria alternata* | [OM236783.1](https://www.ncbi.nlm.nih.gov/nucleotide/OM236783.1?report=genbank&log$=nucltop&blast_rank=2&RID=5E1NCXDT016) | 556 | 100.00% |
| 226 | Third week | KRHD Peak | ON790491 | *Alternaria angustiovoidea* | [OM236751.1](https://www.ncbi.nlm.nih.gov/nucleotide/OM236751.1?report=genbank&log$=nucltop&blast_rank=1&RID=5E25882M016) | 558 | 100.00% |
| 227 | Third week | KRHD Peak | ON790492 | *Cladosporium tenuissimum* | [OM236705.1](https://www.ncbi.nlm.nih.gov/nucleotide/OM236705.1?report=genbank&log$=nucltop&blast_rank=2&RID=5E2GKKWB013) | 528 | 98.82% |
| 228 | Third week | KRHD Peak | ON790493 | *Alternaria solani* | [OM236767.1](https://www.ncbi.nlm.nih.gov/nucleotide/OM236767.1?report=genbank&log$=nucltop&blast_rank=1&RID=5E39JNV1013) | 557 | 100.00% |
| 229 | Third week | KRHD Peak | ON790494 | *Alternaria alternata* | [MW008923.1](https://www.ncbi.nlm.nih.gov/nucleotide/MW008923.1?report=genbank&log$=nucltop&blast_rank=1&RID=5E3H2KE3013) | 544 | 99.81% |
| 230 | Third week | KRHD Peak | ON790495 | *Alternaria compacta* | [OM236750.1](https://www.ncbi.nlm.nih.gov/nucleotide/OM236750.1?report=genbank&log$=nucltop&blast_rank=1&RID=5E49P15H013) | 555 | 99.63% |
| 231 | Third week | KRHD Peak | ON790496 | *Alternaria alternata* | OM237179.1 | 555 | 100.00% |
| 232 | Third week | KRHD Peak | ON790497 | *Alternaria tenuissima* | [MN989220.1](https://www.ncbi.nlm.nih.gov/nucleotide/MN989220.1?report=genbank&log$=nucltop&blast_rank=2&RID=5E53YXMG013) | 535 | 98.29% |
| 233 | Third week | KRHD Peak | ON790498 | *Alternaria alternata* | [OM236934.1](https://www.ncbi.nlm.nih.gov/nucleotide/OM236934.1?report=genbank&log$=nucltop&blast_rank=1&RID=5E5BJHGU013) | 549 | 98.89% |
| 234 | Third week | KRHD Peak | ON790499 | *Alternaria tenuissima* | [ON790499.1](https://www.ncbi.nlm.nih.gov/nucleotide/ON790499.1?report=genbank&log$=nucltop&blast_rank=1&RID=6Y17S1PB016) | 544 | 100.00% |
| 235 | Third week | KRHD Peak | ON790500 | *Alternaria tamaricis* | [MG462836.1](https://www.ncbi.nlm.nih.gov/nucleotide/MG462836.1?report=genbank&log$=nucltop&blast_rank=1&RID=5FM3UWE4016) | 541 | 99.81% |
| 236 | Third week | KRHD Peak | ON790501 | *Alternaria alternata* | [OM237230.1](https://www.ncbi.nlm.nih.gov/nucleotide/OM237230.1?report=genbank&log$=nucltop&blast_rank=2&RID=5FMF68W9013) | 556 | 99.81% |
| 237 | Third week | KRHD Peak | ON790502 | *Alternaria alternata* | [OM236755.1](https://www.ncbi.nlm.nih.gov/nucleotide/OM236755.1?report=genbank&log$=nucltop&blast_rank=1&RID=5K2269HF01R) | 558 | 99.82% |
| 238 | Third week | LRHD Peak | ON790503 | *Cladosporium cladosporioides* | [OM237232.1](https://www.ncbi.nlm.nih.gov/nucleotide/OM237232.1?report=genbank&log$=nucltop&blast_rank=1&RID=5K31TVER013) | 535 | 99.61% |
| 239 | Third week | LRHD Peak | ON790504 | *Alternaria alternata* | [MW008893.1](https://www.ncbi.nlm.nih.gov/nucleotide/MW008893.1?report=genbank&log$=nucltop&blast_rank=2&RID=5K6J586K013) | 551 | 99.44% |
| 240 | Third week | LRHD Peak | ON790505 | *Pseudozyma* sp.  *Moesziomyces antarcticus* | [MN515010.1](https://www.ncbi.nlm.nih.gov/nucleotide/MN515010.1?report=genbank&log$=nucltop&blast_rank=1&RID=5K68H6K901R)  [LC368626.1](https://www.ncbi.nlm.nih.gov/nucleotide/LC368626.1?report=genbank&log$=nucltop&blast_rank=2&RID=5K68H6K901R) | 774  1362 | 99.46%  99.07% |
| 241 | Third week | LRHD Peak | ON790506 | *Alternaria tamaricis* | [OM236768.1](https://www.ncbi.nlm.nih.gov/nucleotide/OM236768.1?report=genbank&log$=nucltop&blast_rank=2&RID=5K5Y1X3B016) | 552 | 100.00% |
| 242 | Third week | LRHD Peak | ON790507 | *Phoma moricola* | [MG462850.1](https://www.ncbi.nlm.nih.gov/nucleotide/MG462850.1?report=genbank&log$=nucltop&blast_rank=1&RID=5K5NPXCZ01R) | 513 | 99.61% |
| 243 | Third week | LRHD Peak | ON790508 | *Alternaria alternata* | [OM237242.1](https://www.ncbi.nlm.nih.gov/nucleotide/OM237242.1?report=genbank&log$=nucltop&blast_rank=1&RID=5K57RHVN01R) | 546 | 99.45% |
| 244 | Third week | LRHD Peak | ON790509 | *Curvularia* sp. | [KP689203.1](https://www.ncbi.nlm.nih.gov/nucleotide/KP689203.1?report=genbank&log$=nucltop&blast_rank=1&RID=7REY8XGW013) | 569 | 99.82% |

**Supplementary Table S3.** Airborne fungal genera and number of strains isolated in each sampling location. WRND = No. 92, Weijin Road, Nankai District, Tianjin, MSRHD = Meteorological Station Road, Heping District, Tianjin, XRHD = No. 2, Xikang Road, Heping District, Tianjin, KRHD = No. 74 Kunming Road, Heping District, Tianjin, LRHD = Lanzhou Road, Heping District, Tianjin.

| Fungal genera | WRND | | MSRHD | | XRHD | | KRHD | | LRHD | | Total | Percentage  (%) |
| --- | --- | --- | --- | --- | --- | --- | --- | --- | --- | --- | --- | --- |
|  | Off-peak | Peak | Off-peak | Peak | Off-peak | Peak | Off-peak | Peak | Off-peak | Peak |  |  |
| *Alternaria* | 1 | 8 | 0 | 12 | 0 | 10 | 5 | 17 | 0 | 9 | 62 | 25.41 |
| *Cladosporium* | 1 | 12 | 2 | 8 | 1 | 1 | 1 | 3 | 5 | 7 | 41 | 16.80 |
| *Coprinellus* | 4 | 0 | 9 | 0 | 4 | 0 | 5 | 0 | 9 | 0 | 31 | 12.70 |
| *Aspergillus* | 0 | 5 | 0 | 2 | 3 | 3 | 0 | 13 | 2 | 1 | 29 | 11.89 |
| *Penicillium* | 1 | 0 | 0 | 0 | 0 | 4 | 3 | 0 | 0 | 0 | 8 | 3.28 |
| *Aureobasidium* | 0 | 0 | 2 | 0 | 2 | 1 | 0 | 0 | 0 | 0 | 5 | 2.05 |
| *Colletotrichum* | 2 | 0 | 1 | 0 | 1 | 0 | 0 | 0 | 0 | 0 | 4 | 1.64 |
| *Fusarium* | 0 | 0 | 0 | 1 | 0 | 0 | 0 | 0 | 3 | 0 | 4 | 1.64 |
| *Paraconiothyrium* | 0 | 0 | 3 | 0 | 0 | 0 | 1 | 0 | 0 | 0 | 4 | 1.64 |
| *Periconia* | 0 | 0 | 0 | 1 | 2 | 0 | 0 | 1 | 0 | 0 | 4 | 1.64 |
| *Stagonosporopsis* | 1 | 0 | 2 | 0 | 0 | 0 | 0 | 0 | 1 | 0 | 4 | 1.64 |
| *Dothideomycetes* | 0 | 0 | 1 | 1 | 1 | 0 | 0 | 0 | 0 | 0 | 3 | 1.23 |
| *Efibula* | 0 | 0 | 2 | 0 | 0 | 0 | 0 | 0 | 1 | 0 | 3 | 1.23 |
| *Filobasidium* | 2 | 0 | 0 | 0 | 0 | 0 | 0 | 0 | 0 | 1 | 3 | 1.23 |
| *Nothophoma* | 0 | 0 | 0 | 1 | 0 | 2 | 0 | 0 | 0 | 0 | 3 | 1.23 |
| *Schizophyllum* | 0 | 0 | 1 | 0 | 0 | 0 | 2 | 0 | 0 | 0 | 3 | 1.23 |
| *Curvularia* | 0 | 0 | 1 | 0 | 0 | 0 | 0 | 0 | 0 | 1 | 2 | 0.82 |
| *Phoma* | 0 | 0 | 0 | 1 | 0 | 0 | 0 | 0 | 0 | 1 | 2 | 0.82 |
| *Setosphaeria* | 0 | 0 | 0 | 0 | 1 | 0 | 1 | 0 | 0 | 0 | 2 | 0.82 |
| *Spencermartinsia* | 1 | 0 | 1 | 0 | 0 | 0 | 0 | 0 | 0 | 0 | 2 | 0.82 |
| *Ascochyta* | 0 | 0 | 1 | 0 | 0 | 0 | 0 | 0 | 0 | 0 | 1 | 0.41 |
| *Ceratobasidium* | 0 | 0 | 1 | 0 | 0 | 0 | 0 | 0 | 0 | 0 | 1 | 0.41 |
| *Cercospora* | 0 | 0 | 0 | 0 | 1 | 0 | 0 | 0 | 0 | 0 | 1 | 0.41 |
| *Coprinopsis* | 0 | 0 | 0 | 0 | 0 | 0 | 0 | 1 | 0 | 0 | 1 | 0.41 |
| *Coriolopsis* | 0 | 0 | 0 | 0 | 1 | 0 | 0 | 0 | 0 | 0 | 1 | 0.41 |
| *Cunninghammyces* | 0 | 0 | 0 | 0 | 1 | 0 | 0 | 0 | 0 | 0 | 1 | 0.41 |
| *Cyphellaceae* | 0 | 0 | 1 | 0 | 0 | 0 | 0 | 0 | 0 | 0 | 1 | 0.41 |
| *Diaporthe* | 0 | 0 | 0 | 0 | 0 | 0 | 0 | 0 | 1 | 0 | 1 | 0.41 |
| *Dothioraceae* | 0 | 0 | 0 | 0 | 1 | 0 | 0 | 0 | 0 | 0 | 1 | 0.41 |
| *Dothiorella* | 0 | 0 | 1 | 0 | 0 | 0 | 0 | 0 | 0 | 0 | 1 | 0.41 |
| *Epicoccum* | 0 | 0 | 0 | 0 | 0 | 0 | 0 | 1 | 0 | 0 | 1 | 0.41 |
| *Eutypella* | 0 | 0 | 0 | 0 | 0 | 0 | 0 | 0 | 1 | 0 | 1 | 0.41 |
| *Gloeophyllum* | 0 | 0 | 0 | 0 | 1 | 0 | 0 | 0 | 0 | 0 | 1 | 0.41 |
| *Hannaella* | 0 | 0 | 0 | 0 | 0 | 0 | 1 | 0 | 0 | 0 | 1 | 0.41 |
| *Moesziomyces* | 0 | 0 | 0 | 0 | 0 | 0 | 0 | 1 | 0 | 0 | 1 | 0.41 |
| *Neoascochyta* | 0 | 0 | 0 | 0 | 1 | 0 | 0 | 0 | 0 | 0 | 1 | 0.41 |
| *Neocamarosporium* | 0 | 0 | 0 | 0 | 0 | 0 | 0 | 1 | 0 | 0 | 1 | 0.41 |
| *Paecilomyces* | 0 | 0 | 0 | 0 | 0 | 1 | 0 | 0 | 0 | 0 | 1 | 0.41 |
| *Phaeosphaeria* | 0 | 0 | 1 | 0 | 0 | 0 | 0 | 0 | 0 | 0 | 1 | 0.41 |
| *Pseudozyma* | 0 | 0 | 0 | 0 | 0 | 0 | 0 | 0 | 0 | 1 | 1 | 0.41 |
| *Russulales* | 0 | 0 | 1 | 0 | 0 | 0 | 0 | 0 | 0 | 0 | 1 | 0.41 |
| *Sporidiobolus* | 0 | 0 | 0 | 0 | 1 | 0 | 0 | 0 | 0 | 0 | 1 | 0.41 |
| *Sporobolomyces* | 0 | 0 | 0 | 0 | 1 | 0 | 0 | 0 | 0 | 0 | 1 | 0.41 |
| *Stagonospora* | 0 | 0 | 1 | 0 | 0 | 0 | 0 | 0 | 0 | 0 | 1 | 0.41 |
| *Talaromyces* | 0 | 0 | 0 | 0 | 0 | 1 | 0 | 0 | 0 | 0 | 1 | 0.41 |

**Supplementary Table S4.** Airborne fungal concentration at each sampling. WRND = No. 92, Weijin Road, Nankai District, Tianjin, MSRHD = Meteorological Station Road, Heping District, Tianjin, XRHD = No. 2, Xikang Road, Heping District, Tianjin, KRHD = No. 74 Kunming Road, Heping District, Tianjin, LRHD = Lanzhou Road, Heping District, Tianjin.

| **Sampling sites** | | **First week**  **‎(CFU/m^3^‎)‎** | **Second week**  **‎(CFU/m^3^‎)‎** | **Third week**  **‎(CFU/m^3^‎)‎** |
| --- | --- | --- | --- | --- |
| **WRND** | Off-peak | 80 | 30 | 20 |
|  | Peak | 110 | 100 | 40 |
| **MSRHD** | Off-peak | 120 | 90 | 110 |
|  | Peak | 90 | 100 | 80 |
| **XRHD** | Off-peak | 110 | 10 | 110 |
|  | Peak | 80 | 10 | 140 |
| **KRHD** | Off-peak | 90 | 60 | 40 |
|  | Peak | 90 | 170 | 120 |
| **LRHD** | Off-peak | 170 | 20 | 40 |
|  | Peak | 120 | 20 | 70 |

**Supplementary Table S5**. Fungal genera distribution at the sampling sites during car traffic off-peak and peak periods.

| **Fungal genera** | **Total strains (off-peak)** | **Total strains (peak)** |
| --- | --- | --- |
| *Alternaria* | 6 | 56 |
| *Ascochyta* | 1 | 0 |
| *Aspergillus* | 5 | 24 |
| *Aureobasidium* | 4 | 1 |
| *Ceratobasidium* | 1 | 0 |
| *Cercospora* | 1 | 0 |
| *Cladosporium* | 10 | 31 |
| *Colletotrichum* | 4 | 0 |
| *Coprinellus* | 31 | 0 |
| *Coprinopsis* | 0 | 1 |
| *Coriolopsis* | 1 | 0 |
| *Cunninghammyces* | 1 | 0 |
| *Curvularia* | 1 | 1 |
| *Cyphellaceae* | 1 | 0 |
| *Diaporthe* | 1 | 0 |
| *Dothideomycetes* | 2 | 1 |
| *Dothioraceae* | 1 | 0 |
| *Dothiorella* | 1 | 0 |
| *Efibula* | 3 | 0 |
| *Epicoccum* | 0 | 1 |
| *Eutypella* | 1 | 0 |
| *Filobasidium* | 2 | 1 |
| *Fusarium* | 3 | 1 |
| *Gloeophyllum* | 1 | 0 |
| *Hannaella* | 1 | 0 |
| *Moesziomyces* | 0 | 1 |
| *Neoascochyta* | 1 | 0 |
| *Neocamarosporium* | 0 | 1 |
| *Nothophoma* | 0 | 3 |
| *Paecilomyces* | 0 | 1 |
| *Paraconiothyrium* | 4 | 0 |
| *Penicillium* | 4 | 4 |
| *Periconia* | 2 | 2 |
| *Phaeosphaeria* | 1 | 0 |
| *Phoma* | 0 | 2 |
| *Pseudozyma* | 0 | 1 |
| *Russulales* | 1 | 0 |
| *Schizophyllum* | 3 | 0 |
| *Setosphaeria* | 2 | 0 |
| *Spencermartinsia* | 2 | 0 |
| *Sporidiobolus* | 1 | 0 |
| *Sporobolomyces* | 1 | 0 |
| *Stagonospora* | 1 | 0 |
| *Stagonosporopsis* | 4 | 0 |
| *Talaromyces* | 0 | 1 |
| **45 genera in total** | **110** | **134** |

**Supplementary Table S6.** Environmental parameters, Temperature (^o^C) and Relative Humidity (%), recorded during each sampling. WRND = No. 92, Weijin Road, Nankai District, Tianjin, MSRHD = Meteorological Station Road, Heping District, Tianjin, XRHD = No. 2, Xikang Road, Heping District, Tianjin, KRHD = No. 74 Kunming Road, Heping District, Tianjin, LRHD = Lanzhou Road, Heping District, Tianjin.

| **Location** | **First week** | | **Second week** | | **Third week** | |
| --- | --- | --- | --- | --- | --- | --- |
|  | **Temperature (^o^C)** | **RH (%)** | **Temperature (^o^C)** | **RH (%)** | **Temperature (^o^C)** | **RH (%)** |
| **WRND** (Off-peak) | 19.4 | 84.2 | 24.8 | 72.0 | 21.6 | 76.7 |
| Peak | 22.0 | 69.4 | 26.8 | 66.7 | 26.8 | 63.1 |
| **MSRHD** (Off-peak) | 19.5 | 88.7 | 25.4 | 70.5 | 20.3 | 81.4 |
| Peak | 22.8 | 65.4 | 27.3 | 66.2 | 25.9 | 66.9 |
| **XRHD** (Off-peak) | 19.5 | 82.7 | 25.4 | 70.5 | 21.9 | 74.3 |
| Peak | 22.4 | 64.0 | 27.3 | 68.6 | 26.6 | 64.6 |
| **KRHD** (Off-peak) | 19.7 | 82.7 | 25.5 | 70.9 | 19.9 | 82.3 |
| Peak | 23.1 | 65.3 | 25.8 | 64.1 | 25.8 | 65.7 |
| **LRHD** (Off-peak) | 19.6 | 83.6 | 25.7 | 70.8 | 19.5 | 82.9 |
| Peak | 22.7 | 64.3 | 25.9 | 64.2 | 26.2 | 66.5 |

**Supplementary Table S7.** The dbRDA Permutation test.

| Name | SumsOfSqs | MeanSqs | F.Models | R2 | Pr(>F) |
| --- | --- | --- | --- | --- | --- |
| RH | 1.983567 | 1.983567 | 6.873553 | 0.197099 | 0.001 |
| T | 1.514966 | 1.514966 | 4.961972 | 0.150536 | 0.001 |
| Groups | 1.653944 | 1.653944 | 5.506686 | 0.164346 | 0.001 |

**Supplementary Figure S1.** Total number of isolated fungal strains from each sampling site during off-peak and peak periods. WRND = No. 92, Weijin Road, Nankai District, Tianjin, MSRHD = Meteorological Station Road, Heping District, Tianjin, XRHD = No. 2, Xikang Road, Heping District, Tianjin, KRHD = No. 74 Kunming Road, Heping District, Tianjin, LRHD = Lanzhou Road, Heping District, Tianjin. Error bars represent SEM.


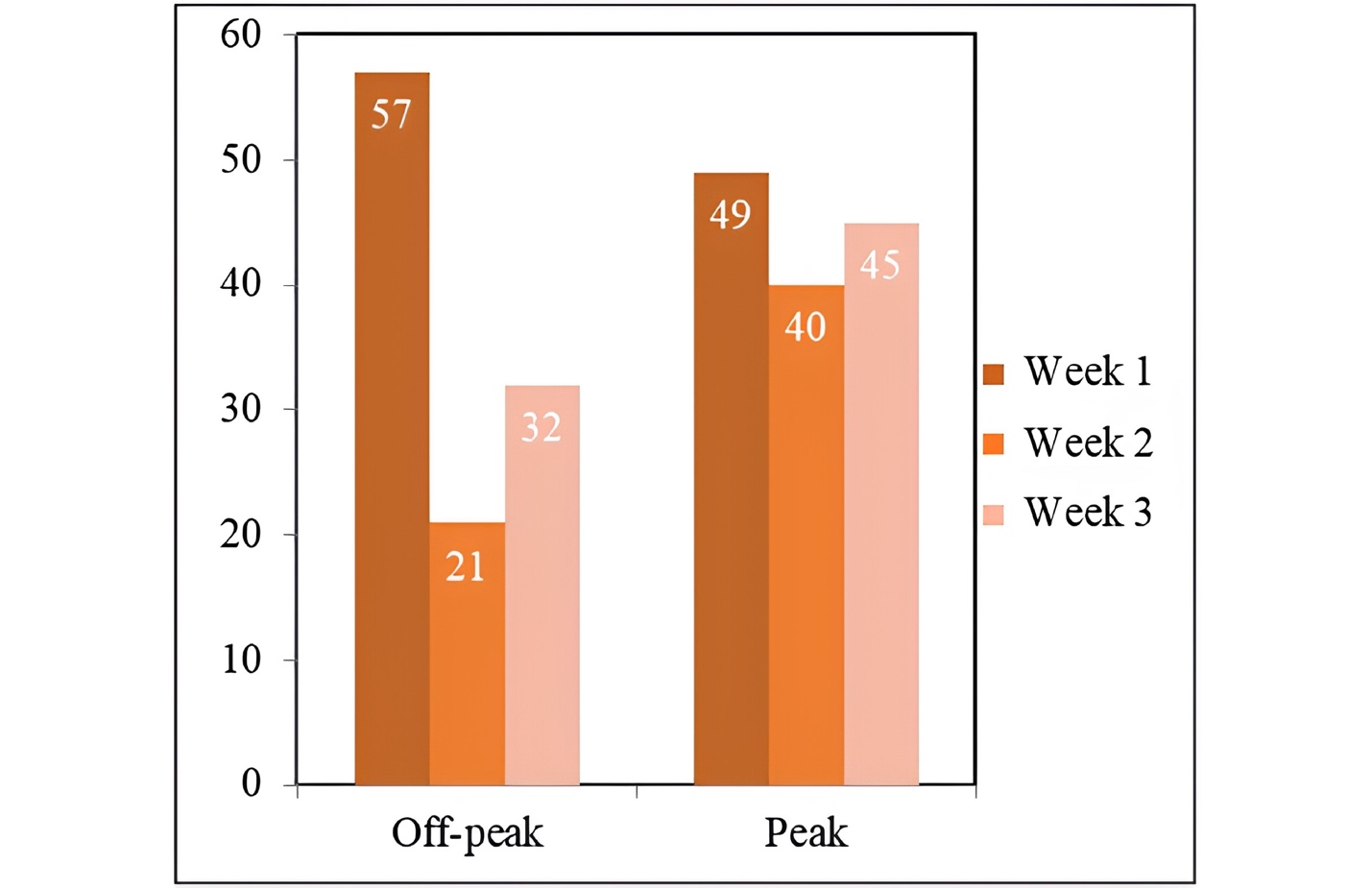


**Supplementary Figure S2.** Total isolated strains in the three sampling weeks at car traffic off-peak and peak periods.

**Supplementary Figure S3.** Fungal concentration at each sampling. WRND = No. 92, Weijin Road, Nankai District, Tianjin, MSRHD = Meteorological Station Road, Heping District, Tianjin, XRHD = No. 2, Xikang Road, Heping District, Tianjin, KRHD = No. 74 Kunming Road, Heping District, Tianjin, LRHD = Lanzhou Road, Heping District, Tianjin.


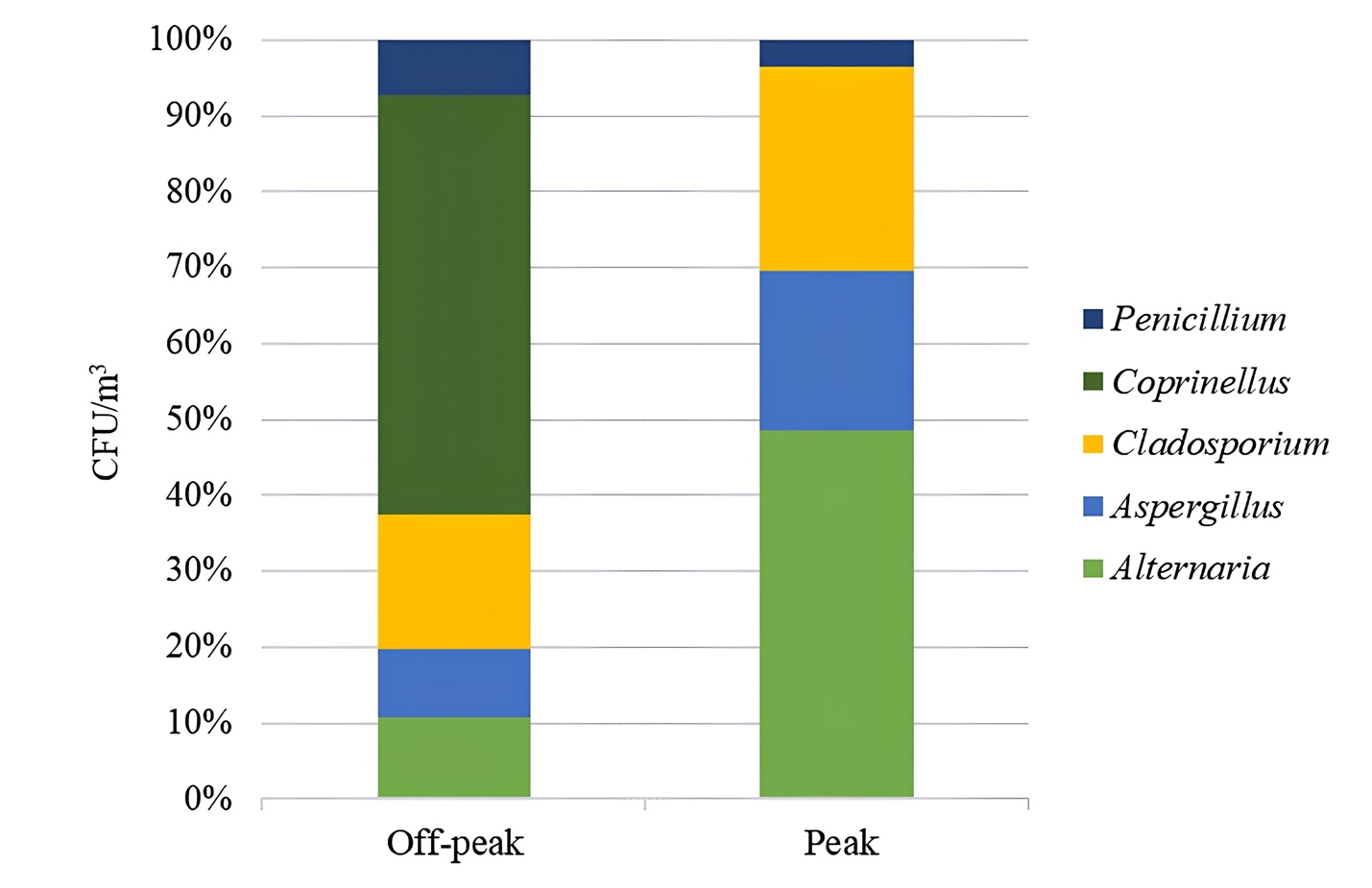


**Supplementary Figure S4.** Relative concentration of the five most abundant fungal genera detected during off-peak and peak hours at the selected traffic junctions.


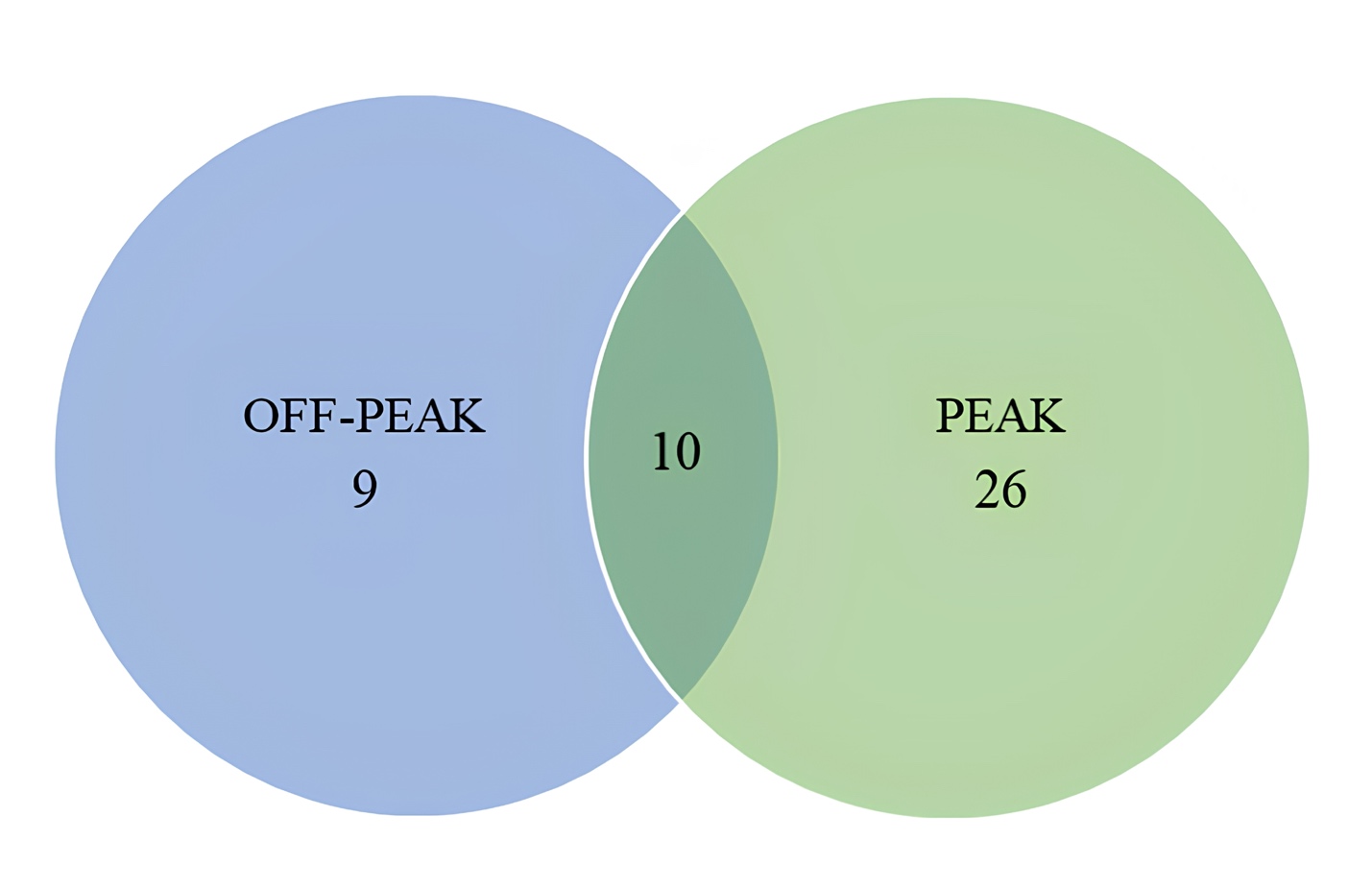


**Supplementary Figure S5.** Total number of fungal genera shared by off-peak and peak traffic periods.


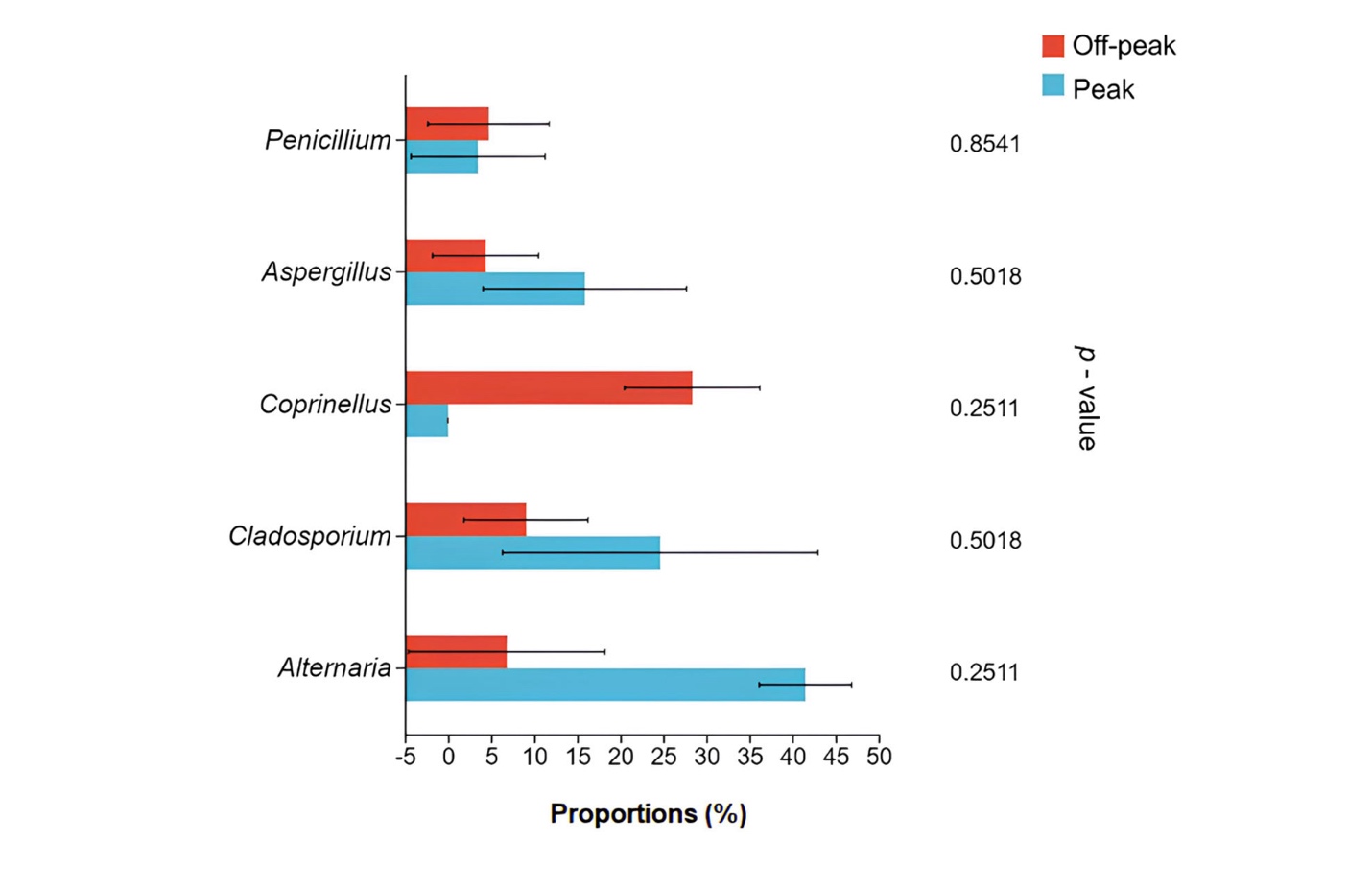


**Supplementary Figure S6.** Variation of five most abundant fungal genera during the two investigated periods (off-peak and peak) based on Wilcoxon rank-sum test. Error bars represent SEM.
